# Increasing presence of Bigg’s killer whales and changing seasonality of Southern Resident killer whales in Washington waters

**DOI:** 10.1371/journal.pone.0350181

**Published:** 2026-06-24

**Authors:** Zoe R. Rand, Laura E. Koehn, Alexis Morrigan, M. Bradley Hanson

**Affiliations:** 1 Quantitative Ecology and Resource Management, University of Washington, Seattle, Washington, United States of America; 2 West Coast Regional Office, National Marine Fisheries Service, National Oceanic and Atmospheric Administration, Seattle, Washington, United States of America; 3 The Whale Museum, Friday Harbor, Washington, United States of America; 4 Northwest Fisheries Science Center, National Marine Fisheries Service, National Oceanic and Atmospheric Administration, Seattle, Washington, United States of America; Texas A&M University, UNITED STATES OF AMERICA

## Abstract

Two ecotypes of killer whales (*Orcinus orca)* occur in the Washington waters of the Salish Sea: endangered, fish-eating Southern Resident killer whales (SRKW), made up of three family groups J-, K-, and L-pods, and marine-mammal-eating Bigg’s killer whales. Although these ecotypes overlap in space, they respond to different ecological processes, face different threats, and have different management priorities. Understanding how their habitat use has changed over time can give us insight into changes in the ecosystem that may be affecting these populations and enable spatially explicit management strategies. Using killer whale detections in Washington waters from 1978–2022, we fit species distribution models to SRKW and Bigg’s killer whales, to understand spatiotemporal trends in killer whale presence. We found that SRKW presence was quite variable, but they were most likely to be present in 2001 (70% average probability of presence) and least likely to be present in 2019 (23% average probability of presence). Bigg’s presence increased over time, from 4% average probability of presence in 1978 to 66% probability of presence in 2022. The decrease in SRKW probability of presence in recent years was most likely driven by K- and L-pods which showed a decline in average annual probability of presence since 2017, while the average annual probability of presence remained high for J-pod. On a seasonal level, SRKW presence has decreased in summer months (June-August) since 2016, while Bigg’s presence has continued to increase in all months. As Bigg’s presence has increased in Washington waters, SRKW and Bigg’s habitat use has increasingly overlapped, especially in the Puget Sound. Since 2011, the probability of Bigg’s presence has increased in the Whidbey Basin while SRKW presence has decreased. Additionally, in October – January, SRKW and Bigg’s have an equal probability of being present throughout the Central Basin. These models can be used to determine optimal times and areas for management actions to limit exposure of anthropogenic disturbances to SRKW and Bigg’s killer whales.

## Introduction

Killer whales (*Orcinus orca*) occurring in inland waters of Washington (part of the Salish Sea) consist of two ecotypes, the Endangered Species Act-listed Southern Resident killer whale (SRKW, *O. o. ater*), which were a fish-eating population of 73 individuals in 2024, and marine-mammal eating Bigg’s killer whales (*O. o. rectipinnus)* [1–3]. SRKWs generally remain in larger social groups which have distinct behaviors and vocal repertoires [4]. Bigg’s, on the other hand, tend to travel in small groups or alone, and roam widely [1]. Although these ecotypes overlap in space, they respond to different ecological processes, face different threats, and have different management priorities. Understanding the differences in their habitat use over time can provide us with information about how the ecosystem might have changed over time and lead to actions to benefit the management of these killer whales, especially in the face of the long-term decline of endangered SRKW from 98 in the 1990s to 73 in 2024 after recovering from substantial removals for the aquarium industry in the 1970s [3].

There can be large inter-annual variability in SRKW arrival time and days present in Washington waters [5,6]. SRKW spend a substantial amount of time in the waterways of the Strait of Georgia, Strait of Juan de Fuca, and Puget Sound except during late winter especially for J-pod [1,5–9]. However, a recent study showed that from 1994–2017 there was a shift in SRKW peak occurrence in the central Salish Sea of 1–5 days later per year [6,10]. This shift in timing follows patterns of shifts in peak occurrence of Fraser River Chinook salmon [6]; a stock of known importance to SRKW diet [11,12]. Similarly, in a recent paper, Stewart et al. [13] showed a decline in all SRKW pods in their core inland summer habitat (north Puget Sound) from 2004 to 2020 and proposed that this shift was related to annual Fraser Chinook salmon returns.

Past studies have shown an increase in occurrence of Bigg’s killer whales in inland waters of Washington over time [14,15]. Houghton et al. [14] showed a significant increase in occurrence of Bigg’s in the Salish Sea between the 1980s/1990s and the 2000s/2010s. Though photo-identification effort for Bigg’s killer whales has been ongoing since 1974, abundance estimates are rare. However, photo-identification studies suggest that populations have been increasing by 3–4% per year since 2010 [16,17]. In addition to increasing population size, one hypothesis for this increase in occurrence is the increase in Bigg’s killer whale prey, mainly pinnipeds, within the region [18]. Previous work to quantify Bigg’s presence in the region have been limited in the time frame considered (7 years or less) [e.g., 15, 19], compared two discrete time frames [e.g., 20] or were limited to a single data source for Bigg’s sightings [e.g., 15].

The Whale Museum’s Sightings Archive [21] contains records of SRKW, Bigg’s, and other marine mammal detections from 1948–2022. These data consist of visual sightings from citizen-scientists, commercial whale-watch operators, dedicated observers, and acoustic detections from hydrophone data. This database provides a uniquely long time series over a broad spatial scale that can be used to detect trends in killer whale occurrence in Washington waters.

Species distribution modeling uses observations of animal occurrence to extrapolate species distributions in space and time, when detection data may be biased due to characteristics associated with the area, time of day, or season. These models are useful for understanding habitat preferences and allow for spatiotemporal predictions [22–24]. Though there are a variety of approaches to these models, a common approach uses presence-only data, a method for generating pseudo-absences, and a generalized linear or generalized linear mixed model (GLMM) framework to get a probability of presence across the spatial region [23–26]. Using statistical models such as these allows for not only the estimation of probability of species presence but also the estimation of underlying uncertainty. Species distribution models are an incredibly flexible framework that can be used for a variety of species and taxa. The models we describe here can provide a framework for estimating distributions of other marine mammals and species where detections are largely opportunistic or seasonally biased.

In this study, we investigate how SRKW and Bigg’s presence has changed in Washington waters over time (1978–2022). We also compare how SRKW and Bigg’s habitat use has changed relative to each other, which may have implications for how these ecotypes interact with each other. To do this, we use species distribution models fit to Bigg’s and SRKW detections from the Whale Museum Archives database. Using a consistent modeling approach for both ecotypes allows us to directly compare results between them. These species distribution models can lead to further insight into causes of shifting killer whale occurrence and benefit the spatial management of threats to SRKW. Notably, these models can help to target or restrict actions in specific times/areas that reduce impacts to SRKW, such as vessel slowdowns (see Quiet Sound: https://quietsound.org/), and federal actions that are required to have ESA consultations.

## Materials and methods

### Killer whale sightings data

The data used in this study consisted of detections from the Whale Museum’s Sightings Network [21]. This database was chosen for its historic value, containing reports that go back over 50 years, and its large scope of both study area and species monitored. These data consist of visual sightings from citizen-scientists, commercial whale-watch operators, dedicated observers, and acoustic detections from hydrophones. Many of the reports housed in the database are difficult to validate on their own, since they were collected from a variety of sighting platforms, observer qualifications, and seasonality. However, summer reports collected by the San Juan County Marine Mammal Stranding Network, Soundwatch, Straitwatch, and data collected from the hydrophone at Lime Kiln State Park are all systematic in their approach and collection and are used to validate other reports. All reports provided to The Whale Museum’s Sightings Archive were processed by the database manager for consistency, accuracy, and completeness before being entered into a Microsoft Access database [see [5]].

Since much of the killer whale presence data comes from opportunistic data sources (citizen science and whale-watch vessel operators) there are inherent biases to the spatial and temporal coverage of these data. For example, more sightings occur closer to populated areas and shorelines; where most people are, not necessarily where there are the most whales. During the off season (fall and winter) and on inclement weather days there is less observation effort. Additionally, there was a noted increase in reports generated after the early 2000s, which is likely due to social media becoming more accessible and widely accepted, rather than an increase in observers [5].

**Fig 1:**
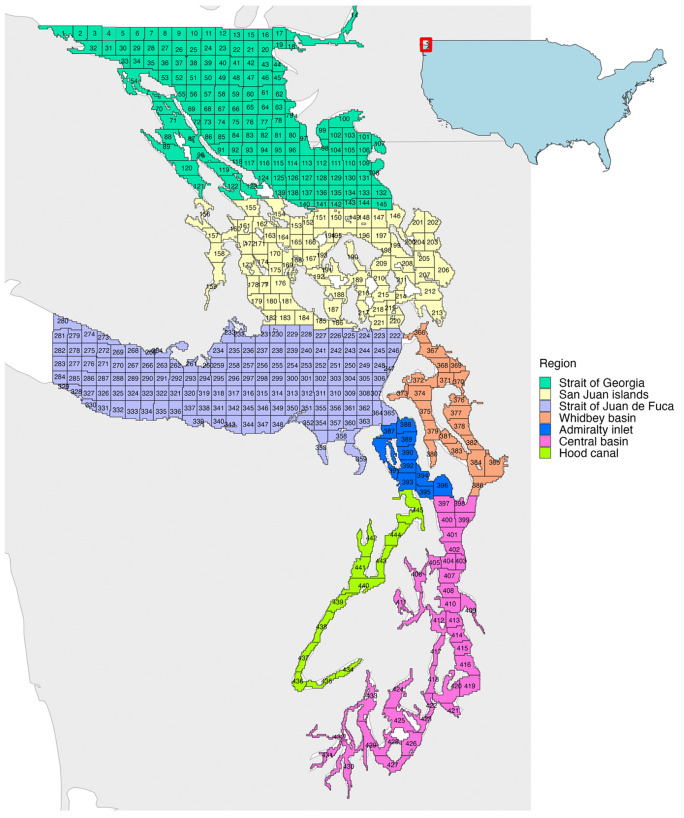
Map of study area including quadrants (rectangles and numbers) and fine-scale regions used to examine habitat use (colors). Made with Natural Earth.

The location data for the Sightings Network originally consisted of descriptions of the area where animals were seen, usually referring to a point on land. During the 1980s, Whale Museum staff designed a system that allowed location data to be assigned to a quadrant, each of which measures approximately 4 by 6 km (Fig 1). Locations for all data points were matched from the original description to an assigned quadrant (Fig 1). Hydrophone detections were ascribed to the quadrant where the hydrophone is located. This system of mapping (location = quadrant) allows for comparison over time [27].

Though there are killer whale visual sightings from as early as 1948, we limited the data to sightings after 1978, since this was the start of dedicated sighting effort for killer whales in the region [e.g., [6]]. The final year of data used in this study was 2022. Detections were summarized by day to account for correlation in sightings within a single day, so that a killer whale “presence” was recorded in each quadrant for each day that there was a visual or acoustic detection of a killer whale in that quadrant. This resulted in a dataset of daily presence of each ecotype in each quadrant. Bigg’s and SRKW detections were treated separately: for a given day and quadrant, there could be a presence recorded for SRKW, a presence recorded for Bigg’s, or presences recorded for both.

### Absence data

To understand the spatiotemporal distribution of killer whales in the region, it is necessary to identify areas and times where killer whales are found (presences) and where they are not found (absences). Visual sightings and acoustic detections represent “presence-only” data sources, where killer whale detections are recorded but no information about killer whale absences or observation effort are recorded. Therefore, it is necessary to create “pseudo-absences” to properly analyze this data. There are many methods for generating pseudo-absences for species distribution modeling, most of which involve sampling a random subset of points from the study area, either throughout the entire spatial extent or in a specified buffer around the detections [28]. However, these approaches do not account for biases in the sampling effort that are present when using opportunistic or citizen-science data [29]. Instead, the use of “target-group” background points, where absences for the species of interest are generated from detections of other similar taxa, have been shown to correct for sampling effort biases [29–31].

Following this method, we used detections of other cetaceans (mainly large baleen whales and porpoises; excluding killer whales) to generate absence data points, under the assumption that all such records would also have reported killer whales if they were present. Like the presence data, we only included detections after 1978, and these were aggregated to daily detections in each quadrant. The same set of absences were used for both SRKW and Bigg’s killer whales. Since these data follow similar patterns of spatial and temporal bias in sighting effort, they can control for some of this bias, allowing the model to better predict actual killer whale habitat use rather than sampling effort.

The process for detecting and reporting acoustic observations of cetaceans differs from visual sightings. The ability to detect different species of cetaceans on a hydrophone depends on the frequency of sound produced by the species and the recording frequency range of the hydrophone. Additionally, the distance from which an animal can be heard from a hydrophone depends on the depth, frequency and source level of the species vocalization, as well as environmental conditions and background noise levels. Species may produce sounds for different reasons and during different behavioral states. Therefore, it is possible that the assumptions made about the pseudo-absences above do not apply to acoustic detections. As a sensitivity, additional models were run without the acoustic data and more details about this sensitivity test can be found in the appendix (S1 Data in S1 File).

### SRKW pod data

SRKWs have unique social groups, or “pods”, which generally travel together and have unique behaviors and vocal repertoires [4]. These pods are generally designated as “J”, “K”, and “L”, with individuals assigned unique numbers (e.g., J16). Since all individual SRKWs are known, it is usually possible to identify SRKW detections to pod-level. Therefore, to examine SRKW presence on the pod-level, SRKW detections were also assigned to pods. Confirmed pod identifications were used as listed in the Whale Museum Archives [21]. The presence of an individual from any of the pods was recorded as a detection for that pod, except for L87, who tends to travel with other pods, and therefore if only L87 was present, this was not counted as the presence of any of the pods. Additionally, for some detections of SRKW it was not possible to confirm pod identities, so, in these cases, notes were included about possible pods. These notes were used to assign pod identities for detections without confirmed pod identities.

Individual detections of each pod were aggregated by day and quadrant, like the full SRKW and Bigg’s datasets, resulting in a dataset for each pod. SRKW detections of one pod were assumed to be absences for the other pods. For example, if only J-pod was seen in quadrant *i* on day *t*, then a 1 was recorded in the J-pod dataset on that day and quadrant, and a 0 was recorded for that day and quadrant for K- and L-pods. No other cetacean detections were used as absences for the pod-specific dataset. Additionally, if it was not possible to identify a SRKW detection to the pod-level, then this detection was not used for the pod-specific SRKW dataset (not even as a 0).

### Species distribution model

Models were fit to predict the probability of killer whale presence (*p*) in a given quadrant (*s*) month (*m*) and year (*y*) using year and month as fixed effects and a gaussian random field to estimate underlying spatial variation ($\omega_{s}$) and spatiotemporal variation ($\epsilon_{s,\;t}$).


$$\begin{array}{c} ogit\left(p_{s,m,t}\right)={year}_{t}+s\left({Month}_{m}\right)+s\left({Month}_{m},\;{year}_{t}\right)+\omega_{s}+\;\epsilon_{s,\;t}\; \end{array}$$ (1)

A logit-link function was used so that the probability of presence was estimated to be between 0 and 1 and the predicted probability was linked to data with a binomial distribution. A cyclic smoother on month, *s(),* was used to allow for non-linear effects of months across all years, and a factor smooth by year was used to allow for annual deviations around this effect [e.g., [32]]. The basis dimension, *k = 9*, was chosen for all models. The spatiotemporal field was assumed to be independent between years (allowing the mean spatial distribution across all months in a year to be unique). The models were fit with the package *sdmTMB* [33], which combines the Stochastic Partial Differential Equation [34, 35] with maximum likelihood estimation using Template Model Builder [36] in R v. 4.4.1 [37]. A barrier mesh was built to prevent model predictions on land using a map of the Puget Sound and central Salish Sea which was developed by the Northwest Fisheries Science Center using data obtained from the National Oceanographic and Atmospheric Administration’s (NOAA) geophysical data center and the British Columbia Marine Conservation Analysis project team. The mesh was built using a cutoff distance of 10 km and generated 145 vertices.

Models were fit separately for SRKW and Bigg’s, following the structure above. Additional models for each SRKW pod were run for just the SRKW detections following the same structure as the above models. For the pod-specific models, only detections of other SRKW pods were used as absences and therefore the estimated probability of presence in these models should be interpreted as conditional on SRKW presence. Convergence was checked by confirming that the Hessian matrix was positive definite and that all log likelihood gradients were <0.001. Residuals were checked using qq-plots and by visually examining the spatial and temporal residuals for patterns. The ability of the model to distinguish between species presence and absence was assessed using the True Skill Statistic (TSS) [38,39]. TSS was calculated for each model, and for a model run with 3-fold cross validation (with randomly selected folds) with the TSS calculated as the average across folds.

Predictions of the probability of SRKW or Bigg’s presence were converted to annual averages for the entire study region, and monthly probabilities for the Puget Sound (quadrants 366–445, Fig 1) and central Salish Sea (quadrants 1–363, Fig 1). To do this, the models were used to estimate the probability of presence for each year, month, and quadrant for both SRKW and Bigg’s. These were then averaged over the number of quadrants that made up each region (total study region: n = 445, central Salish Sea: n = 367, Puget Sound: n = 80). For the annual averages, the monthly probabilities were also averaged over months. These estimates were constructed using 1000 simulations from the joint-precision matrix and the averaging was done for each simulation. This allows for the estimated uncertainty in the model to be propagated through to the predictions. The 2.5%, 50% and 97.5% quantiles of these averages were used to get an estimate and measure of uncertainty.

### Space-use overlap between SRKW and Bigg’s

To compare fine-scale space use between SRKW and Bigg’s, the models were used to calculate relative probabilities of presence of each ecotype in each year, month, and quadrant. The predicted relative probability (*R)* of SRKW presence to Bigg’s presence was calculated as:


$$\begin{array}{c} {\hat{R}}_{SRKW}=\;\frac{{\hat{p}}_{SRKW}}{{\hat{p}}_{SRKW}+\;{\hat{p}}_{TKW}} \end{array}$$ (2)

where ${\hat{p}}_{SRKW}\;$ represents the predicted probability of SRKW presence and ${\hat{p}}_{TKW}\;$ represents the predicted probability of Bigg’s presence. This results in a metric ranging from 0 to 1, where values closer to 0 indicates lower probabilities of SRKW presence and higher probabilities of Bigg’s presence, and values closer to 1 indicate higher probabilities of SRKW presence and lower probabilities of Bigg’s presence, and a value of 0.5 indicates equal probability of SRKW and Bigg’s presence. Like the average probabilities above, relative probabilities of the SRKW presence were calculated for each year, month, and quadrant using 1000 simulations from the joint-precision matrix from each model and then using the 2.5%, 50% and 97.5% quantiles of these relative probabilities to get an estimate and measure of uncertainty.

## Results

Since 1978, 10,548 days with SRKW detections and 8,910 days with Bigg’s detections were recorded. 60% of all reported Bigg’s detections and 65% of reported SRKW detections occurred in May through September. 45% of SRKW and 31% of Bigg’s detections were in the San Juan Islands. As a sensitivity, models were also run excluding acoustic detections. This resulted in 127 fewer days of SRKW presence (2.0% of days with SRKW presence) and 68 fewer days of Bigg’s presence (1.6% of days with Bigg’s presence; S1 Data in S1 File).

Species distribution models which accounted for annual and seasonal variation in species presence (eqn. 1) were fit separately to SRKW and Bigg’s killer whales to predict the probability of presence for each quadrant (Fig 1) in each month and year (S1 and S2 Tables, S1-S4 Figs in S1 File). All TSS scores were positive (S3 Table in S1 File), indicating that the models did better at classifying species presence and absence than random allocation [38].

**Fig 2:**
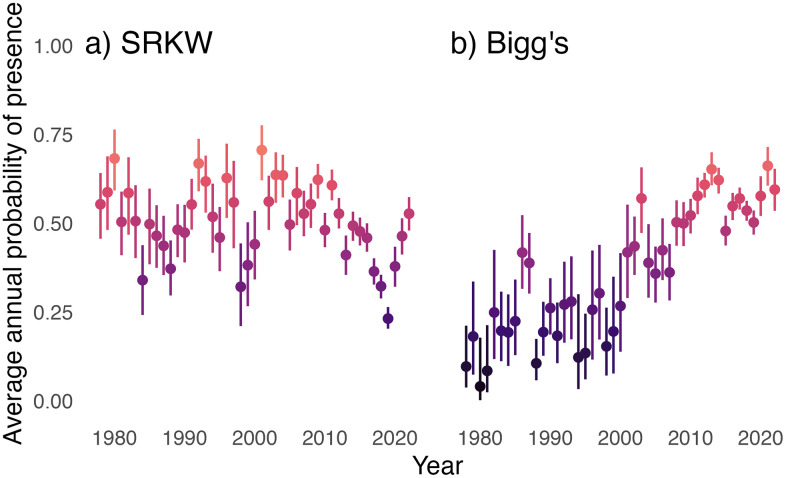
Average predicted probability of presence (points) and 95% prediction intervals (errorbars) across all quadrants in each year for SRKW (a) and Bigg’s (b). Cooler colors represent probabilities closer to 0 and warmer colors represent probabilities closer to 1.

The annual predicted probability of SRKW presence from the species distribution model, averaged across the entire study region, ranged from 0.232 (95% interval: 0.203–0.264) to 0.706 (0.621–0.776) between 1978 and 2022 with the lowest probabilities of presence in 1984, 1998, 2018 and 2019 and the highest in 1980, 1992 and 2001. The annual probability of SRKW presence had high variation and did not demonstrate a clear overall trend (Fig 2a). The average annual predicted probability of Bigg’s presence ranged from 0.041 (0.002–0.178) to 0.662 (0.606–0.715) between 1978 and 2022, demonstrating an increasing trend over time with the lowest predicted probability in 1980 and the highest in 2021(Fig 2b). The sensitivity models without acoustic data resulted in a < 3% difference in almost all average predicted probabilities of presence across years and ecotypes. Additionally, the confidence intervals of these predictions from both models overlapped, suggesting there was no difference in the predicted annual pattern when this data was included or excluded (S1 Data in S1 File).

**Fig 3:**
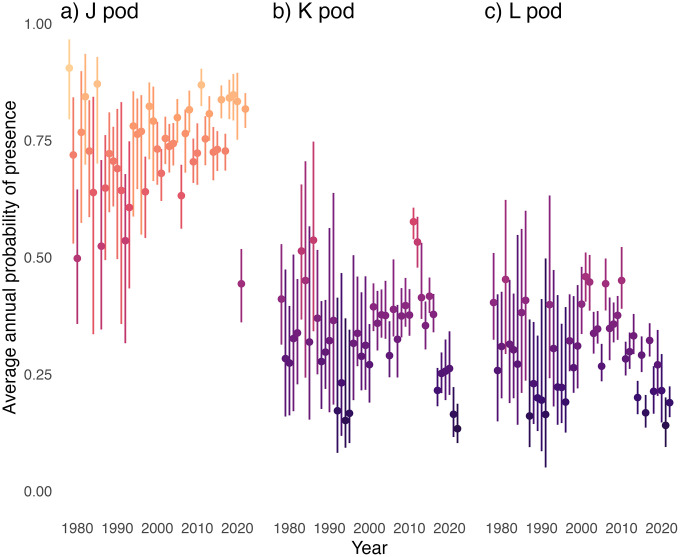
Average predicted probability of presence (points) and 95% prediction intervals (errorbars) across all quadrants in each year for J-pod (a), K-pod (b) and L-pod (c). Probability of presence of pods are conditional on SRKW presence (Figure 2). Cooler colors represent probabilities closer to 0 and warmer colors represent probabilities closer to 1.

For SRKW, 5,124 days with J-pod detections, 3,2424 days with K-pod detections, and 3,061 days with L-pod detections were recorded between 1978 and 2022. Additional pod-specific models were run which describe the conditional probability of each pod being present given SRKW presence (S4 Table in S1 File). The average annual predicted probability of J-pod presence (given the presence of SRKW) was high, ranging from 0.443 (0.361–0.518) in 2021 to 0.905 (0.795–0.966) in 1978 (Fig 3a). K-pod was present less often, ranging from 0.134 (0.103–0.187) in 2022 to 0.576 (0.539–0.606) in 2011 and L-pod was the least likely to be present, ranging from 0.141 (0.094–0.200) in 2021 to 0.459 (0.404–0.510) in 2001. Both K- and L-pods demonstrated a decrease in probability of presence in the most recent years (since 2017–2018; Fig 3b-c).

**Fig 4:**
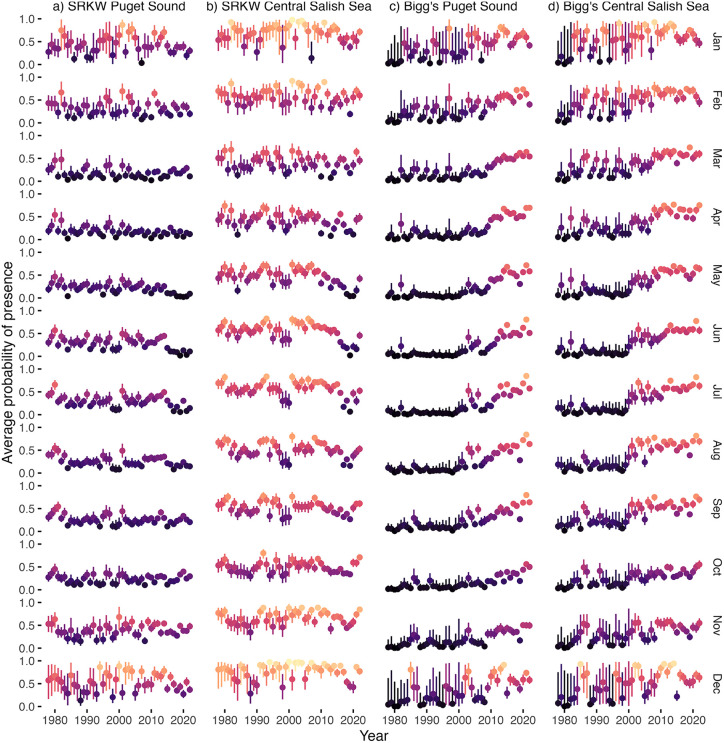
Average monthly probability (points) and 95% prediction intervals (errorbars) of SRKW (a, b) and Bigg’s (c, d) presence in the Puget Sound (a, c) and Central Salish Sea (b, d). Warmer colors represent values closer to 1 and cooler colors represent values closer to 0.

SRKW are generally less likely to be present in the Puget Sound than the central Salish Sea in all months (Fig 4). In most years, SRKW presence was highest in December-January in both regions with predicted probabilities greater than 0.7 in 37 years in the central Salish Sea and 11 years in the Puget Sound (Fig 4a-b). SRKW had a predicted probability of presence that ranged from 0.22–0.29 in the Puget Sound in the summer (June-August) from 1978–2015, but since 2016 SRKW have been less likely to be present in summer months (average 0.13 from June-August; Fig 4a). SRKW were also less likely to be present in the central Salish Sea since 2016, except in 2022 (Fig 4b). Bigg’s presence in both the central Salish Sea and the Puget Sound show an increasing trend in all months since the early 2000s (Fig 4c-d). In the Puget Sound, predicted probabilities of Bigg’s presence from 1978–2001 were generally lower than SRKW probabilities, but since 2002, Bigg’s have had higher probabilities of presence than SRKW. Similarly, in the central Salish Sea, Bigg’s probabilities of presence were initially lower than SRKW but have been approaching comparable levels to SRKW probabilities since 2001(Fig 4). The sensitivity models without acoustic data resulted in a < 5% difference in almost all average predicted probabilities of presence across months, years, regions, and ecotypes. Additionally, the confidence intervals of the predictions from both models overlapped, suggesting there was no difference in predicted monthly patterns when this data was included or excluded (S1 File).

**Fig 5:**
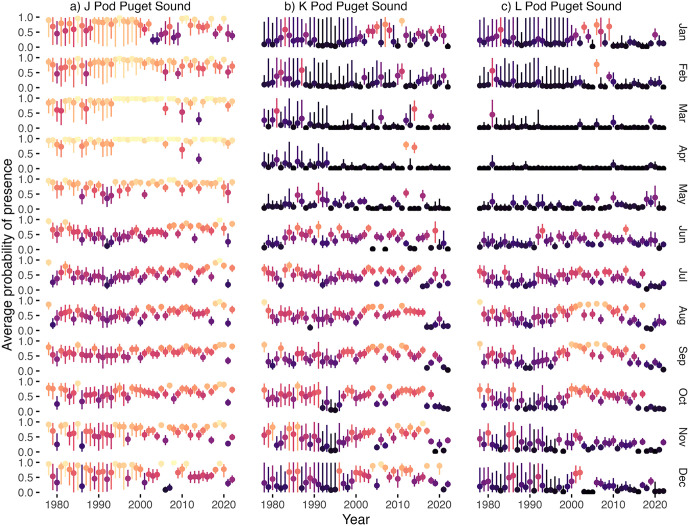
Average monthly probability (points) and 95% prediction intervals (errorbars) of J, K, and L-pods (a, b, c respectively) presence in the Puget Sound. Warmer colors represent values closer to 1 and cooler colors represent values closer to 0.

Pod-specific models demonstrate seasonal differences in SRKW presence in the Puget Sound, with J-pod presence likely year-round (Fig 5a). K-pod and L-pod, however, have higher probabilities of presence June – January, with the highest probabilities of presence in June – October (Fig 5b-c). K and L-pod presence have decreased in all months since 2016 (Fig 5b-c).

**Fig 6:**
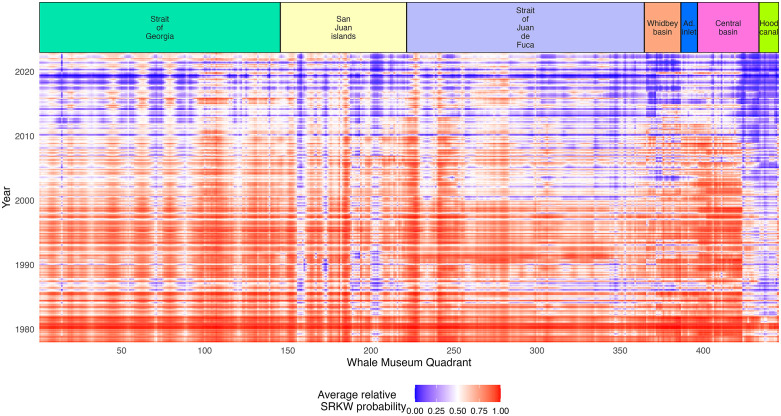
Average relative probability of SRKW and Bigg’s presence by quadrant. Red represents probabilities closest to 100% that a killer whale is a SRKW, blue represents probabilities closest to 0% that a killer whale is a SRKW and therefore reflects 100% probabilities of Bigg’s presence. White represents a 50% probability of either a SRKW or a Bigg’s presence (given killer whale presence). Colored labels represent the regional location of the quadrants (see Fig 1).

As Bigg’s presence has increased in Washington waters, habitat overlap between SRKW and Bigg’s has increased in all regions (Fig 6). In the Puget Sound, Bigg’s have largely been present in the Hood Canal since the late 1980s, however, since 2001, Bigg’s presence has increased in the Whidbey Basin, Admiralty Inlet, and the Central Basin. SRKW and Bigg’s presence largely overlaps in the Admiralty Inlet and Central Basin, while Bigg’s presence seems to have supplanted SRKW presence in Whidbey Basin since 2015 (Fig 6).

**Fig 7:**
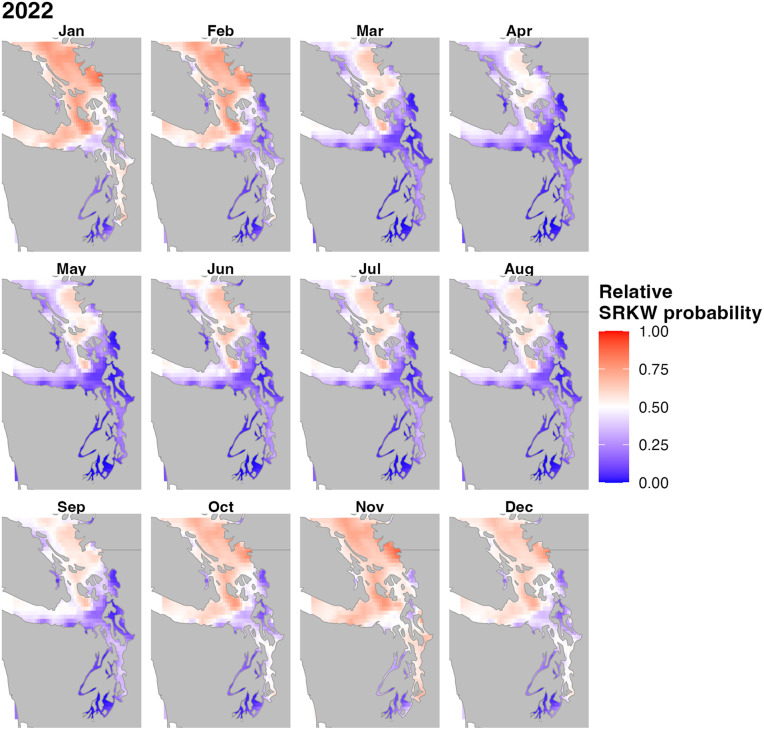
Predicted relative probability of SRKW and Bigg’s presence in study region in 2022. Red represents probabilities closest to 100% that a killer whale is a SRKW, blue represents probabilities closest to 0% that a killer whale is a SRKW and therefore reflects 100% probabilities of Bigg’s presence. White represents a 50% chance of either a SRKW or a Bigg’s presence (given killer whale presence). Made with Natural Earth.

In the final year of our study, 2022, SRKW and Bigg’s were predicted to have equal probabilities of presence in parts of the Puget Sound, especially the Central Basin during the fall and winter (Oct-Jan). Bigg’s probabilities of presence in the central Salish Sea remained relatively lower during this period. During the spring and summer (Mar-Sep), Bigg’s were more likely to be present in the Puget Sound, while SRKW remained closer to the Washington coastline in the central Salish Sea (Fig 7).

## Discussion

In this study we built species distribution models for SRKW and Bigg’s killer whales in Washington waters using a decades-long sightings database held by The Whale Museum [21]. We found that Bigg’s presence has greatly increased in the region since 1978 (Fig 2, Fig 4). This is consistent with other recent work [14,15], which has also found increasing Bigg’s presence in Washington waters and our study illustrates that this trend is continuing. It has been hypothesized that this is due to increasing pinniped abundance in the region, a main source of prey for Bigg’s.

Patterns in SRKW presence in the region, however, are more complicated. As is shown in Ettinger et al. [6] and Stewart et al. [13], we found declines in SRKW presence in the Puget Sound, particularly in the summer months (June-August; Fig 4). However, the overall presence across years has not declined (Fig 2). Research has pointed to shifts in presence due to changes in Chinook prey of SRKW [6,13]. There is also some evidence for competition for prey between SRKW and the Northern Resident killer whales that overlap with SRKW which could be influencing presence [40,41]. High abundances of pink salmon has been linked to reduced body condition, increased mortality and lower reproductive rates for SRKW [42] which could also be a factor in SRKW presence depending on the regional distribution of pink salmon. Other threats to SRKW include disturbance from vessels and noise [43,44] and it is unknown how these may be impacting SRKW presence especially as vessels increase in the Salish Sea and Puget Sound [45].

This continued overall presence of SRKW is largely driven by J-pod, since K- and L-pod presence has declined since 2016 (Fig 5). There is some evidence that specific Chinook stocks are of variable importance to the different pods [12] and diet varies across pods [46] which could help to explain variation in presence between the pods. Social and cultural learning likely also play an important role in killer whale habitat use and foraging behavior. The strength of social cohesion between members of each pod may be influenced by environmental factors, such as prey availability, as well as population size [47]. Furthermore, individuals and particular matrilines may also play an important role in social cohesion [48]. Differences in the social cohesion among pods may contribute to the variation in presence seen in our study.

The distributions in 2022 show high probabilities of SRKW presence compared to Bigg’s presence around the San Juan Islands and the West Coast of British Columbia year-round (Fig 7). This matches known SRKW hotspots [5,8] and supports certain recent fisheries area closures in Canada to reduce competition for SRKW (https://www.pac.dfo-mpo.gc.ca/fm-gp/mammals-mammiferes/whales-baleines/srkw-measures-mesures-ers-eng.html). In the U.S., the west side of San Juan Island, especially around Salmon Bank, can be a popular fishing area [49]. A voluntary no-go zone for boaters is in place off the west side of San Juan (https://wdfw.wa.gov/species-habitats/at-risk/species-recovery/orca/rule-making) and a new 1000 yard distance rule is in place for boaters to maintain distance from SRKW [50]. As SRKWs continue to use this area throughout the year, these protections are likely important for this endangered species when they are near San Juan Island.

As Bigg’s presence has increased in the region, the likelihood of both SRKW and Bigg’s presence in the same month has increased, leading to greater habitat overlap especially in the Puget Sound (Fig 6). For instance, in 2022 there were similar probabilities of presence for both SRKW and Bigg’s in the Central Basin and Admiralty inlet (Fig 6-7). It is important to note that since our model operates on a monthly scale, we cannot determine whether Bigg’s and SRKW are sharing habitats at the same time or interacting with each other. Finer-scale temporal separation may be occurring, with different ecotypes using the same area at different times of day or different days of the month. Anecdotal evidence suggests that Bigg’s and SRKW may avoid each other [15,19]. However, these patterns show that both SRKW and Bigg’s are increasingly present in the same areas, leading to a higher likelihood of fine-scale habitat overlap and interaction. Habitat overlap between these two ecotypes may suggest increased foraging by Bigg’s on the pinniped populations (though no recent diet data for Bigg’s currently exist) which compete with SRKW for salmonid prey [18]. This may positively impact SRKW, by releasing more salmonid prey for SRKW consumption. However, it may also displace SKRW from their primary habitat, leading to interaction with other fish-eating killer whales (such as Northern resident killer whales) [51].

These models are the first to employ the decades-long sightings database held by The Whale Museum for both SRKW and Bigg’s. By using a similar modelling framework for both ecotypes, without ecotype-specific covariates, we were able to create models that were easily comparable and led to similar predictions. However, these models could be modified in the future to include prey-specific and other environmental covariates to improve predictions for specific ecotypes and killer whale presence overall in the future. This would require more spatially explicit datasets on salmon and pinniped abundance in the region than are currently available [52,53]. A future joint model of both SRKW and Bigg’s, incorporating prey-specific covariates, could allow for a deeper understanding of interactions between these two ecotypes, the effects of Bigg’s predation on pinnipeds, and resulting pinniped consumption of salmon.

In addition, environmental covariates could be added to the model as salmon demographics may be linked to changing climate. Quinn and Losee [54] found that Salish Sea residential salmonids have declined and are smaller than non-resident fish, and this could be tied to changes in environmental conditions. Southern Residents prefer larger salmonids [55] and therefore this may cause a decrease in their occurrence in winter months when larger, non-residential salmon have migrated outside of Salish Sea to coastal waters [54]. Other research has shown a decline in Chinook size and age across the U.S. West Coast [56], therefore, less biomass for Southern Residents to consume. Additionally, Puget Sound Chinook were found to have high exposure and sensitivity to changing environmental conditions compared to certain other salmonid populations [57], and preliminary results suggest temperature and salinity are increasing in Puget Sound while dissolved oxygen is declining [58]. As previously mentioned, Stewart et al. [13] found that shifts in Southern Resident timing in Salish sea may be related to Fraser Chinook abundance, and preliminary analyses suggest these fish are vulnerable to environmental conditions [59]. Fraser Chinook have also experienced a decline in size at age that may be linked to environmental variables [60] and more lipid-rich Fraser runs have declined [61], which the authors note could be tied to ocean conditions.

The distribution models presented here are useful to resource managers dealing with both Bigg’s and SRKW. Understanding killer whale presence in Puget Sound can provide important information about the role of top predators in the health of the Puget Sound ecosystem. The models we present here could be used to calculate the residence time of each ecotype in the region, which alongside other ecological indicators, could be used to monitor progress towards the Washington state recovery goal of having thriving species and food webs in the Puget Sound [62].

These models can also be useful to managers in the recovery of SRKW, especially addressing actions that interact with the three main threats to SRKW – prey availability, noise/vessel disturbance, and contaminants. The Salish Sea region is home to more than nine million people in Washington and British Columbia, major shipping lanes, and other anthropogenic activity [63]. Under Section 7 of the Endangered Species Act, federal agencies must ensure their actions are not likely to jeopardize the existence of a listed species or adversely modify critical habitat of listed species. Federal actions include in-water construction, military activities, fisheries, dredging, offshore energy development, aquaculture, etc. Our killer whale distribution models can be used to determine the optimal times and areas for actions to occur to minimize likely exposure to SRKW. Results can also be used to identify times or areas to prioritize actions that may benefit SRKW – e.g., volunteer vessel restriction such as those led by Quiet Sound (https://quietsound.org/). Actions such as a state vessel distance requirement of 1000 yards and a voluntary no-go zone off San Juan Island (https://wdfw.wa.gov/fishing/management/mpa/whale-protection-zone) help limit interactions between fisheries and SRKW but these models could help to fine-tune times and areas where overlap between SRKW and salmon fisheries should be minimized.

For Bigg’s, although they are not listed under the ESA, they are protected under the Marine Mammal Protection Act which prohibits the harassment, hunting, capturing, collecting, or killing of marine mammals and permits need to be acquired for activities which may harm marine mammals. The Bigg’s model can help to inform when and where permits are needed and areas and times where actions can occur with limited likelihood of harming Bigg’s. Therefore, these models can help management protect species through limiting overlap between whales and other maritime economies while still allowing these activities to continue.

## Supporting information

S1 File S1 Fig. Spatial field for SRKW distribution model. Made with Natural Earth. S2 Fig. Spatiotemporal field in each year for SRKW distribution model. Made with Natural Earth. S3 Fig. Spatial field for Bigg’s distribution model. Made with Natural Earth. S4 Fig. Spatiotemporal field in each year for Bigg’s distribution model. Made with Natural Earth.S1 Table. Parameter estimates for SRKW distribution model, with a fixed effect of year as a factor, a cyclic smoother for non-linear effects of month across all years, and a factor smooth for annual deviations around this effect, and independent and identically distributed spatiotemporal fields for each year. S2 Table. Parameter estimates for Bigg’s distribution model, with a fixed effect of year as a factor, a cyclic smoother for non-linear effects of month across all years, and a factor smooth for annual deviations around this effect, and independent and identically distributed spatiotemporal fields for each year. S3 Table. True Skill Statistics (TSS) for each model with both the full dataset and one with 3-fold cross validation where the TSS is averaged across all three folds. TSS > 0 indicates that the model is performing better at distinguishing species presence/absence than random allocation and TSS **=** 1 would indicate perfect allocation. S4 Table. Parameter estimates for SRKW pod-specific distribution model. S1 Data. Sensitivity analysis: Models without hydrophone data.
(ZIP)
