# Supplementary material for: Increasing presence of Bigg’s killer whales and changing seasonality of Southern Resident killer whales in Washington waters
Source: PLoS One. 2026 Jun 24;21(6):e0350181. doi: 10.1371/journal.pone.0350181 (PMC13293393; doi:10.1371/journal.pone.0350181)
Supplement: S1 File — S1 Fig. Spatial field for SRKW distribution model. Made with Natural Earth. S2 Fig. Spatiotemporal field in each year for SRKW distribution model. Made with Natural Earth. S3 Fig. Spatial field for Bigg’s distribution model. Made with Natural Earth. S4 Fig. Spatiotemporal field in each year for Bigg’s distribution model. Made with Natural Earth.S1 Table. Parameter estimates for SRKW distribution model, with a fixed effect of year as a factor, a cyclic smoother for non-linear effects of month across all years, and a factor smooth for annual deviations around this effect, and independent and identically distributed spatiotemporal fields for each year. S2 Table. Parameter estimates for Bigg’s distribution model, with a fixed effect of year as a factor, a cyclic smoother for non-linear effects of month across all years, and a factor smooth for annual deviations around this effect, and independent and identically distributed spatiotemporal fields for each year. S3 Table. True Skill Statistics (TSS) for each model with both the full dataset and one with 3-fold cross validation where the TSS is averaged across all three folds. TSS > 0 indicates that the model is performing better at distinguishing species presence/absence than random allocation and TSS = 1 would indicate perfect allocation. S4 Table. Parameter estimates for SRKW pod-specific distribution model. S1 Data. Sensitivity analysis: Models without hydrophone data. (ZIP) [file pone.0350181.s001.zip › Supporting Information 3/S4 Table.docx]

**S4 Table. Parameter estimates for SRKW pod-specific distribution model.**

| **Model** | **Parameter** | **Description** | **estimate** | **SE** | **2.5%** | **97.5%** |
| --- | --- | --- | --- | --- | --- | --- |
| **J pod** | Year: 1978 | Fixed effect of year | 3.26 | 0.58 | 2.12 | 4.41 |
|  | Year: 1979 | Fixed effect of year | 0.55 | 0.62 | -0.67 | 1.77 |
|  | Year: 1980 | Fixed effect of year | -0.49 | 0.52 | -1.50 | 0.53 |
|  | Year: 1981 | Fixed effect of year | 1.53 | 0.52 | 0.50 | 2.56 |
|  | Year: 1982 | Fixed effect of year | 2.54 | 0.66 | 1.25 | 3.83 |
|  | Year: 1983 | Fixed effect of year | 0.80 | 0.48 | -0.15 | 1.74 |
|  | Year: 1984 | Fixed effect of year | 0.33 | 0.65 | -0.95 | 1.60 |
|  | Year: 1985 | Fixed effect of year | 2.55 | 0.82 | 0.94 | 4.15 |
|  | Year: 1986 | Fixed effect of year | -0.43 | 0.57 | -1.54 | 0.68 |
|  | Year: 1987 | Fixed effect of year | 0.60 | 0.48 | -0.34 | 1.54 |
|  | Year: 1988 | Fixed effect of year | 0.57 | 0.48 | -0.38 | 1.52 |
|  | Year: 1989 | Fixed effect of year | 0.76 | 0.44 | -0.09 | 1.62 |
|  | Year: 1990 | Fixed effect of year | 0.47 | 0.59 | -0.68 | 1.62 |
|  | Year: 1991 | Fixed effect of year | 0.16 | 0.79 | -1.40 | 1.71 |
|  | Year: 1992 | Fixed effect of year | -1.01 | 0.61 | -2.20 | 0.18 |
|  | Year: 1993 | Fixed effect of year | -0.16 | 0.56 | -1.26 | 0.93 |
|  | Year: 1994 | Fixed effect of year | 1.06 | 0.56 | -0.03 | 2.16 |
|  | Year: 1995 | Fixed effect of year | 0.84 | 0.49 | -0.12 | 1.81 |
|  | Year: 1996 | Fixed effect of year | 0.96 | 0.56 | -0.15 | 2.07 |
|  | Year: 1997 | Fixed effect of year | -0.04 | 0.39 | -0.79 | 0.72 |
|  | Year: 1998 | Fixed effect of year | 1.34 | 0.42 | 0.52 | 2.16 |
|  | Year: 1999 | Fixed effect of year | 1.26 | 0.42 | 0.43 | 2.09 |
|  | Year: 2000 | Fixed effect of year | 0.15 | 0.40 | -0.63 | 0.94 |
|  | Year: 2001 | Fixed effect of year | 0.29 | 0.35 | -0.38 | 0.97 |
|  | Year: 2002 | Fixed effect of year | 1.23 | 0.38 | 0.48 | 1.98 |
|  | Year: 2003 | Fixed effect of year | 1.20 | 0.37 | 0.48 | 1.92 |
|  | Year: 2004 | Fixed effect of year | 0.76 | 0.33 | 0.10 | 1.42 |
|  | Year: 2005 | Fixed effect of year | 1.55 | 0.37 | 0.82 | 2.28 |
|  | Year: 2006 | Fixed effect of year | 0.27 | 0.37 | -0.45 | 0.99 |
|  | Year: 2007 | Fixed effect of year | 1.51 | 0.38 | 0.76 | 2.25 |
|  | Year: 2008 | Fixed effect of year | 1.90 | 0.42 | 1.08 | 2.72 |
|  | Year: 2009 | Fixed effect of year | 0.80 | 0.35 | 0.12 | 1.48 |
|  | Year: 2010 | Fixed effect of year | 1.65 | 0.39 | 0.89 | 2.42 |
|  | Year: 2011 | Fixed effect of year | 2.34 | 0.39 | 1.57 | 3.10 |
|  | Year: 2012 | Fixed effect of year | 1.08 | 0.35 | 0.39 | 1.77 |
|  | Year: 2013 | Fixed effect of year | 0.83 | 0.38 | 0.08 | 1.59 |
|  | Year: 2014 | Fixed effect of year | 1.40 | 0.35 | 0.71 | 2.09 |
|  | Year: 2015 | Fixed effect of year | 0.87 | 0.34 | 0.20 | 1.54 |
|  | Year: 2016 | Fixed effect of year | 1.78 | 0.36 | 1.07 | 2.48 |
|  | Year: 2017 | Fixed effect of year | 0.57 | 0.37 | -0.16 | 1.30 |
|  | Year: 2018 | Fixed effect of year | 2.32 | 0.38 | 1.58 | 3.06 |
|  | Year: 2019 | Fixed effect of year | 3.52 | 0.58 | 2.38 | 4.67 |
|  | Year: 2020 | Fixed effect of year | 1.74 | 0.44 | 0.87 | 2.60 |
|  | Year: 2021 | Fixed effect of year | -1.03 | 0.41 | -1.84 | -0.22 |
|  | Year: 2022 | Fixed effect of year | 1.79 | 0.36 | 1.09 | 2.49 |
|  | sds(Month) | Factor smooth of month | – | 0.73 | – | – |
|  | sds(Month, Year) | Factor smooth of month and year (1) | – | 20.1 | – | – |
|  | sds(Month,Year) | Factor smooth of month and year (2) | – | 6.84 | – | – |
|  | sds(Month, Year) | Factor smooth of month and year (3) | – | <0.01 | – | – |
|  | range | Matérn range | 85.26 | 8.10 | 70.76 | 102.72 |
|  | $\sigma_{\omega}$ | marginal standard deviation of the spatial field | 7.97 | 1.43 | 5.61 | 11.33 |
|  | $\sigma_{\varepsilon}$ | marginal standard deviation of spatiotemporal field | 8.41 | 0.94 | 6.76 | 10.47 |
| **K pod** | Year: 1978 | Fixed effect of year | 0.09 | 0.55 | -1.00 | 1.18 |
|  | Year: 1979 | Fixed effect of year | -1.12 | 0.58 | -2.25 | 0.02 |
|  | Year: 1980 | Fixed effect of year | -1.25 | 0.47 | -2.17 | -0.32 |
|  | Year: 1981 | Fixed effect of year | -1.17 | 0.43 | -2.02 | -0.32 |
|  | Year: 1982 | Fixed effect of year | -0.99 | 0.54 | -2.05 | 0.08 |
|  | Year: 1983 | Fixed effect of year | -0.04 | 0.43 | -0.89 | 0.81 |
|  | Year: 1984 | Fixed effect of year | 0.08 | 0.73 | -1.35 | 1.50 |
|  | Year: 1985 | Fixed effect of year | -0.92 | 0.86 | -2.60 | 0.76 |
|  | Year: 1986 | Fixed effect of year | 0.90 | 0.72 | -0.51 | 2.31 |
|  | Year: 1987 | Fixed effect of year | -0.65 | 0.44 | -1.52 | 0.21 |
|  | Year: 1988 | Fixed effect of year | -1.33 | 0.53 | -2.36 | -0.29 |
|  | Year: 1989 | Fixed effect of year | -1.06 | 0.39 | -1.83 | -0.30 |
|  | Year: 1990 | Fixed effect of year | -0.86 | 0.62 | -2.08 | 0.36 |
|  | Year: 1991 | Fixed effect of year | -0.47 | 0.86 | -2.16 | 1.21 |
|  | Year: 1992 | Fixed effect of year | -2.27 | 0.58 | -3.40 | -1.14 |
|  | Year: 1993 | Fixed effect of year | -1.71 | 0.58 | -2.84 | -0.58 |
|  | Year: 1994 | Fixed effect of year | -3.21 | 1.23 | -5.62 | -0.79 |
|  | Year: 1995 | Fixed effect of year | -2.33 | 0.81 | -3.93 | -0.74 |
|  | Year: 1996 | Fixed effect of year | -0.63 | 0.53 | -1.66 | 0.41 |
|  | Year: 1997 | Fixed effect of year | -0.45 | 0.35 | -1.13 | 0.24 |
|  | Year: 1998 | Fixed effect of year | -0.73 | 0.35 | -1.42 | -0.04 |
|  | Year: 1999 | Fixed effect of year | -0.69 | 0.37 | -1.42 | 0.03 |
|  | Year: 2000 | Fixed effect of year | -1.50 | 0.38 | -2.24 | -0.75 |
|  | Year: 2001 | Fixed effect of year | -0.43 | 0.31 | -1.04 | 0.19 |
|  | Year: 2002 | Fixed effect of year | -0.25 | 0.33 | -0.90 | 0.40 |
|  | Year: 2003 | Fixed effect of year | -0.10 | 0.34 | -0.76 | 0.56 |
|  | Year: 2004 | Fixed effect of year | -1.24 | 0.38 | -1.99 | -0.50 |
|  | Year: 2005 | Fixed effect of year | -1.21 | 0.33 | -1.86 | -0.57 |
|  | Year: 2006 | Fixed effect of year | 0.13 | 0.34 | -0.53 | 0.79 |
|  | Year: 2007 | Fixed effect of year | -2.16 | 0.38 | -2.92 | -1.41 |
|  | Year: 2008 | Fixed effect of year | -0.48 | 0.35 | -1.16 | 0.20 |
|  | Year: 2009 | Fixed effect of year | -0.22 | 0.31 | -0.83 | 0.39 |
|  | Year: 2010 | Fixed effect of year | -0.28 | 0.35 | -0.96 | 0.39 |
|  | Year: 2011 | Fixed effect of year | 0.78 | 0.34 | 0.11 | 1.46 |
|  | Year: 2012 | Fixed effect of year | -0.17 | 0.32 | -0.80 | 0.46 |
|  | Year: 2013 | Fixed effect of year | -0.59 | 0.39 | -1.35 | 0.17 |
|  | Year: 2014 | Fixed effect of year | -1.17 | 0.34 | -1.83 | -0.51 |
|  | Year: 2015 | Fixed effect of year | -0.37 | 0.32 | -0.99 | 0.25 |
|  | Year: 2016 | Fixed effect of year | -0.54 | 0.32 | -1.18 | 0.09 |
|  | Year: 2017 | Fixed effect of year | -2.40 | 0.39 | -3.16 | -1.65 |
|  | Year: 2018 | Fixed effect of year | -2.11 | 0.34 | -2.78 | -1.45 |
|  | Year: 2019 | Fixed effect of year | -1.27 | 0.45 | -2.16 | -0.39 |
|  | Year: 2020 | Fixed effect of year | -1.90 | 0.47 | -2.83 | -0.97 |
|  | Year: 2021 | Fixed effect of year | -2.45 | 0.49 | -3.40 | -1.49 |
|  | Year: 2022 | Fixed effect of year | -3.28 | 0.40 | -4.07 | -2.49 |
|  | sds(Month) | Factor smooth of month | – | 0.61 | – | – |
|  | sds(Month, Year) | Factor smooth of month and year (1) | – | 48.16 | – | – |
|  | sds(Month,Year) | Factor smooth of month and year (2) | – | 13.23 | – | – |
|  | sds(Month, Year) | Factor smooth of month and year (3) | – | <0.01 | – | – |
|  | range | Matérn range | 104.94 | 10.58 | 86.12 | 127.87 |
|  | $\sigma_{\omega}$ | marginal standard deviation of the spatial field | 4.92 | 1.05 | 3.24 | 7.47 |
|  | $\sigma_{\varepsilon}$ | marginal standard deviation of spatiotemporal field | 8.14 | 0.98 | 6.43 | 10.32 |
| **L pod** | Year: 1978 | Fixed effect of year | 0.40 | 0.59 | -0.76 | 1.56 |
|  | Year: 1979 | Fixed effect of year | -1.36 | 0.61 | -2.55 | -0.16 |
|  | Year: 1980 | Fixed effect of year | -1.53 | 0.56 | -2.62 | -0.44 |
|  | Year: 1981 | Fixed effect of year | -0.44 | 0.51 | -1.44 | 0.56 |
|  | Year: 1982 | Fixed effect of year | -1.32 | 0.59 | -2.48 | -0.15 |
|  | Year: 1983 | Fixed effect of year | -1.86 | 0.56 | -2.95 | -0.76 |
|  | Year: 1984 | Fixed effect of year | -1.38 | 0.77 | -2.88 | 0.13 |
|  | Year: 1985 | Fixed effect of year | -0.56 | 0.66 | -1.85 | 0.72 |
|  | Year: 1986 | Fixed effect of year | -0.67 | 0.62 | -1.88 | 0.54 |
|  | Year: 1987 | Fixed effect of year | -2.56 | 0.63 | -3.80 | -1.32 |
|  | Year: 1988 | Fixed effect of year | -1.63 | 0.55 | -2.71 | -0.54 |
|  | Year: 1989 | Fixed effect of year | -2.27 | 0.49 | -3.24 | -1.30 |
|  | Year: 1990 | Fixed effect of year | -2.33 | 0.61 | -3.51 | -1.14 |
|  | Year: 1991 | Fixed effect of year | -3.17 | 1.20 | -5.53 | -0.82 |
|  | Year: 1992 | Fixed effect of year | -0.56 | 0.58 | -1.70 | 0.59 |
|  | Year: 1993 | Fixed effect of year | -1.07 | 0.62 | -2.30 | 0.15 |
|  | Year: 1994 | Fixed effect of year | -1.79 | 0.65 | -3.06 | -0.51 |
|  | Year: 1995 | Fixed effect of year | -1.73 | 0.51 | -2.74 | -0.72 |
|  | Year: 1996 | Fixed effect of year | -2.37 | 0.65 | -3.66 | -1.09 |
|  | Year: 1997 | Fixed effect of year | -0.58 | 0.41 | -1.39 | 0.22 |
|  | Year: 1998 | Fixed effect of year | -1.20 | 0.54 | -2.25 | -0.15 |
|  | Year: 1999 | Fixed effect of year | -1.12 | 0.44 | -1.98 | -0.26 |
|  | Year: 2000 | Fixed effect of year | 0.13 | 0.45 | -0.75 | 1.01 |
|  | Year: 2001 | Fixed effect of year | 0.42 | 0.37 | -0.30 | 1.15 |
|  | Year: 2002 | Fixed effect of year | 0.33 | 0.40 | -0.45 | 1.11 |
|  | Year: 2003 | Fixed effect of year | -0.84 | 0.41 | -1.63 | -0.04 |
|  | Year: 2004 | Fixed effect of year | -0.54 | 0.39 | -1.31 | 0.24 |
|  | Year: 2005 | Fixed effect of year | -1.28 | 0.40 | -2.07 | -0.49 |
|  | Year: 2006 | Fixed effect of year | 0.12 | 0.38 | -0.62 | 0.87 |
|  | Year: 2007 | Fixed effect of year | -1.37 | 0.39 | -2.14 | -0.60 |
|  | Year: 2008 | Fixed effect of year | 0.06 | 0.41 | -0.75 | 0.87 |
|  | Year: 2009 | Fixed effect of year | -0.21 | 0.37 | -0.94 | 0.52 |
|  | Year: 2010 | Fixed effect of year | 0.02 | 0.39 | -0.75 | 0.79 |
|  | Year: 2011 | Fixed effect of year | -0.51 | 0.39 | -1.28 | 0.25 |
|  | Year: 2012 | Fixed effect of year | -0.74 | 0.37 | -1.48 | -0.01 |
|  | Year: 2013 | Fixed effect of year | -0.14 | 0.41 | -0.94 | 0.67 |
|  | Year: 2014 | Fixed effect of year | -1.61 | 0.38 | -2.36 | -0.86 |
|  | Year: 2015 | Fixed effect of year | -1.23 | 0.36 | -1.95 | -0.52 |
|  | Year: 2016 | Fixed effect of year | -2.46 | 0.42 | -3.27 | -1.64 |
|  | Year: 2017 | Fixed effect of year | -0.34 | 0.40 | -1.13 | 0.45 |
|  | Year: 2018 | Fixed effect of year | -2.10 | 0.40 | -2.89 | -1.31 |
|  | Year: 2019 | Fixed effect of year | -2.12 | 0.44 | -2.97 | -1.26 |
|  | Year: 2020 | Fixed effect of year | -2.45 | 0.50 | -3.42 | -1.47 |
|  | Year: 2021 | Fixed effect of year | -2.72 | 0.52 | -3.75 | -1.69 |
|  | Year: 2022 | Fixed effect of year | -2.29 | 0.37 | -3.01 | -1.56 |
|  | sds(Month) | Factor smooth of month | – | 1.18 | – | – |
|  | sds(Month, Year) | Factor smooth of month and year (1) | – | 25.49 | – | – |
|  | sds(Month,Year) | Factor smooth of month and year (2) | – | 13.12 | – | – |
|  | sds(Month, Year) | Factor smooth of month and year (3) | – | <0.01 | – | – |
|  | range | Matérn range | 97.47 | 8.93 | 81.45 | 116.65 |
|  | $\sigma_{\omega}$ | marginal standard deviation of the spatial field | 7.59 | 1.35 | 5.35 | 10.75 |
|  | $\sigma_{\varepsilon}$ | marginal standard deviation of spatiotemporal field | 9.27 | 0.99 | 7.52 | 11.41 |
