# Supplementary material for: Increasing presence of Bigg’s killer whales and changing seasonality of Southern Resident killer whales in Washington waters
Source: PLoS One. 2026 Jun 24;21(6):e0350181. doi: 10.1371/journal.pone.0350181 (PMC13293393; doi:10.1371/journal.pone.0350181)
Supplement: S1 File — S1 Fig. Spatial field for SRKW distribution model. Made with Natural Earth. S2 Fig. Spatiotemporal field in each year for SRKW distribution model. Made with Natural Earth. S3 Fig. Spatial field for Bigg’s distribution model. Made with Natural Earth. S4 Fig. Spatiotemporal field in each year for Bigg’s distribution model. Made with Natural Earth.S1 Table. Parameter estimates for SRKW distribution model, with a fixed effect of year as a factor, a cyclic smoother for non-linear effects of month across all years, and a factor smooth for annual deviations around this effect, and independent and identically distributed spatiotemporal fields for each year. S2 Table. Parameter estimates for Bigg’s distribution model, with a fixed effect of year as a factor, a cyclic smoother for non-linear effects of month across all years, and a factor smooth for annual deviations around this effect, and independent and identically distributed spatiotemporal fields for each year. S3 Table. True Skill Statistics (TSS) for each model with both the full dataset and one with 3-fold cross validation where the TSS is averaged across all three folds. TSS > 0 indicates that the model is performing better at distinguishing species presence/absence than random allocation and TSS = 1 would indicate perfect allocation. S4 Table. Parameter estimates for SRKW pod-specific distribution model. S1 Data. Sensitivity analysis: Models without hydrophone data. (ZIP) [file pone.0350181.s001.zip › Supporting Information 3/S1 File.docx]

**S1 File. Sensitivity analysis: Models without hydrophone data**

It is possible that our assumptions made about pseudo-absences do not apply to the acoustic detections included in the model (see methods in main text) therefore we ran the same models as described in the paper but excluding any detections from hydrophone data. This excluded 4794 detections of SRKW and 615 detections of Bigg’s (6.5% of total detections). This resulted in a dataset with 127 fewer days of SRKW presence (2.0% of SRKW positive days) and 68 fewer days of Bigg’s presence (1.6% of Bigg’s positive days). Though there were a few hydrophone detections of SRKW in the 1980s, most hydrophone detections for both ecotypes occurred after 2008 (Fig1). No hydrophone detections were included in the dataset of other cetacean sightings used to create the pseudo-absences, so the pseudo-absence dataset is the same for these models as for the models presented in the main text.


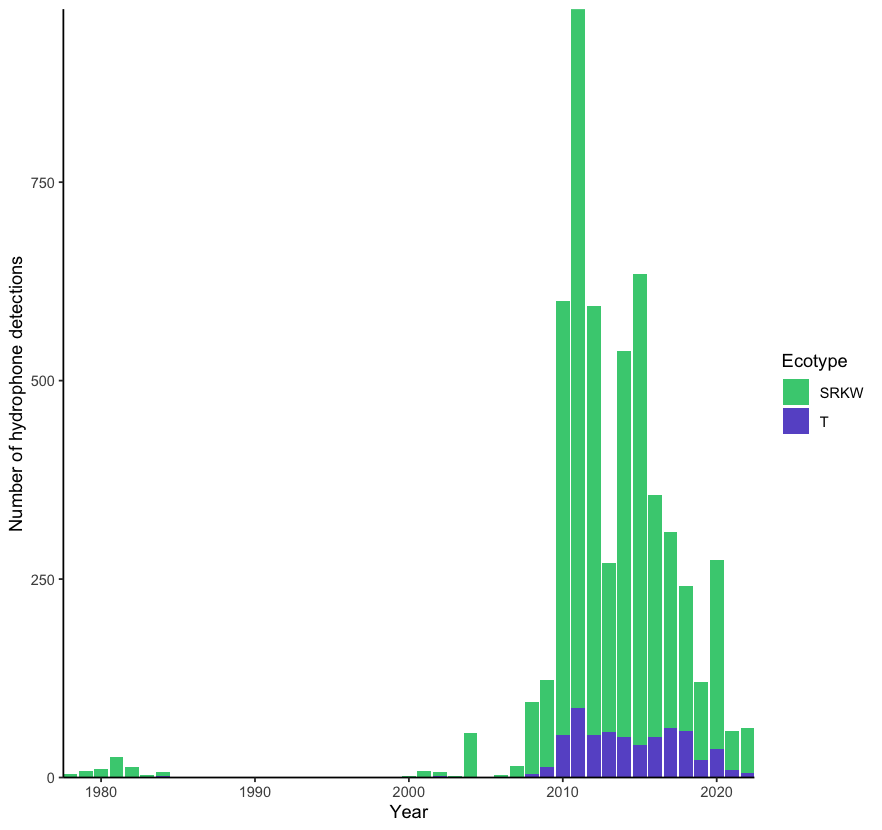


**Fig 1.** Number of detections from hydrophones in each year for SRKW (green) and Bigg’s (blue).

The models without the hydrophone data resulted in different parameter estimates for the fixed effects of year in almost all years (Tables 1 and 2). However, the random effect parameter estimates were largely the same and differed by only about 10% in the model of SRKW and 1% in the model of Bigg’s. This resulted in very similar predictions over space and time between the two models for both SRKW and Bigg’s (Fig 2-3). Therefore, we believe that our decision to include the hydrophone data did not have a significant influence on the results.

**Table 1. Parameter estimates for SRKW distribution model without hydrophone data and percent difference in estimated parameter value from model with all data sources (see Table S1 for full parameter estimates).**

| **Parameter** | **Description** | **estimate** | **SE** | **estimate including hydrophone data** | **% difference in estimates** |
| --- | --- | --- | --- | --- | --- |
| Year: 1978 | Fixed effect of year | -0.81 | 0.42 | -0.83 | 2.73 |
| Year: 1979 | Fixed effect of year | -0.96 | 0.48 | -0.84 | 12.38 |
| Year: 1980 | Fixed effect of year | -0.03 | 0.44 | 0.18 | 282.39 |
| Year: 1981 | Fixed effect of year | -1.18 | 0.44 | -1.35 | 13.81 |
| Year: 1982 | Fixed effect of year | -1.21 | 0.51 | -0.63 | 63.27 |
| Year: 1983 | Fixed effect of year | -1.55 | 0.46 | -1.00 | 42.95 |
| Year: 1984 | Fixed effect of year | -3.29 | 0.48 | -2.87 | 13.60 |
| Year: 1985 | Fixed effect of year | -1.97 | 0.50 | -1.68 | 15.58 |
| Year: 1986 | Fixed effect of year | -1.95 | 0.47 | -1.64 | 17.63 |
| Year: 1987 | Fixed effect of year | -1.88 | 0.44 | -1.70 | 10.34 |
| Year: 1988 | Fixed effect of year | -2.48 | 0.45 | -1.78 | 32.78 |
| Year: 1989 | Fixed effect of year | -1.97 | 0.42 | -1.52 | 26.06 |
| Year: 1990 | Fixed effect of year | -1.52 | 0.44 | -1.21 | 22.16 |
| Year: 1991 | Fixed effect of year | -1.88 | 0.44 | -1.79 | 5.10 |
| Year: 1992 | Fixed effect of year | -0.98 | 0.46 | -0.62 | 45.54 |
| Year: 1993 | Fixed effect of year | -0.97 | 0.45 | -0.77 | 22.51 |
| Year: 1994 | Fixed effect of year | -1.96 | 0.52 | -1.53 | 24.62 |
| Year: 1995 | Fixed effect of year | -2.12 | 0.49 | -1.87 | 13.01 |
| Year: 1996 | Fixed effect of year | -1.13 | 0.52 | -0.78 | 36.60 |
| Year: 1997 | Fixed effect of year | -1.11 | 0.55 | -1.14 | 2.84 |
| Year: 1998 | Fixed effect of year | -2.80 | 0.55 | -2.82 | 0.87 |
| Year: 1999 | Fixed effect of year | -2.98 | 0.58 | -2.85 | 4.35 |
| Year: 2000 | Fixed effect of year | -2.26 | 0.55 | -2.46 | 8.40 |
| Year: 2001 | Fixed effect of year | -0.37 | 0.48 | 0.39 | 7147.83 |
| Year: 2002 | Fixed effect of year | -1.37 | 0.47 | -0.98 | 33.65 |
| Year: 2003 | Fixed effect of year | -1.27 | 0.45 | -0.78 | 47.50 |
| Year: 2004 | Fixed effect of year | -1.26 | 0.45 | -0.69 | 57.73 |
| Year: 2005 | Fixed effect of year | -2.11 | 0.43 | -1.48 | 35.25 |
| Year: 2006 | Fixed effect of year | -1.33 | 0.43 | -0.78 | 51.28 |
| Year: 2007 | Fixed effect of year | -1.60 | 0.44 | -1.16 | 31.75 |
| Year: 2008 | Fixed effect of year | -1.77 | 0.44 | -1.20 | 38.21 |
| Year: 2009 | Fixed effect of year | -1.40 | 0.41 | -0.78 | 56.40 |
| Year: 2010 | Fixed effect of year | -2.01 | 0.41 | -2.18 | 7.87 |
| Year: 2011 | Fixed effect of year | -1.49 | 0.41 | -1.22 | 19.29 |
| Year: 2012 | Fixed effect of year | -1.63 | 0.37 | -1.53 | 6.53 |
| Year: 2013 | Fixed effect of year | -2.28 | 0.42 | -2.28 | 0.07 |
| Year: 2014 | Fixed effect of year | -1.50 | 0.39 | -1.42 | 5.59 |
| Year: 2015 | Fixed effect of year | -2.05 | 0.38 | -1.63 | 22.77 |
| Year: 2016 | Fixed effect of year | -2.45 | 0.38 | -2.00 | 20.00 |
| Year: 2017 | Fixed effect of year | -3.10 | 0.39 | -2.62 | 16.61 |
| Year: 2018 | Fixed effect of year | -3.25 | 0.39 | -2.84 | 13.27 |
| Year: 2019 | Fixed effect of year | -4.08 | 0.40 | -3.87 | 5.40 |
| Year: 2020 | Fixed effect of year | -3.01 | 0.41 | -2.68 | 11.82 |
| Year: 2021 | Fixed effect of year | -3.05 | 0.40 | -2.79 | 8.86 |
| Year: 2022 | Fixed effect of year | -2.43 | 0.38 | -2.03 | 17.82 |
| range | Matérn range | 47.71 | 2.64 | 49.89 | 4.46 |
| $\sigma_{\omega}$ | marginal standard deviation of the spatial field | 13.63 | 1.50 | 15.05 | 9.89 |
| $\sigma_{\varepsilon}$ | marginal standard deviation of spatiotemporal field | 7.62 | 0.51 | 8.29 | 8.39 |

**Table 2**. **Parameter estimates for Bigg’s distribution models without hydrophone data and percent difference in estimated parameter value from model with all data sources (see Table S1 for full parameter estimates).**

| **Parameter** | **Description** | **estimate** | **SE** | **estimate including hydrophone data** | **% difference in estimates** |
| --- | --- | --- | --- | --- | --- |
| Year: 1978 | Fixed effect of year | -4.06 | 0.85 | -3.59 | 12.25 |
| Year: 1979 | Fixed effect of year | -2.41 | 0.73 | -2.90 | 18.21 |
| Year: 1980 | Fixed effect of year | -5.62 | 1.31 | -6.04 | 7.29 |
| Year: 1981 | Fixed effect of year | -3.88 | 0.80 | -4.33 | 10.94 |
| Year: 1982 | Fixed effect of year | -1.66 | 0.69 | -2.01 | 19.30 |
| Year: 1983 | Fixed effect of year | -2.81 | 0.68 | -3.17 | 12.04 |
| Year: 1984 | Fixed effect of year | -3.71 | 0.73 | -4.12 | 10.54 |
| Year: 1985 | Fixed effect of year | -2.91 | 0.75 | -3.26 | 11.19 |
| Year: 1986 | Fixed effect of year | -0.91 | 0.56 | -1.23 | 29.93 |
| Year: 1987 | Fixed effect of year | -1.48 | 0.56 | -1.94 | 27.25 |
| Year: 1988 | Fixed effect of year | -3.00 | 0.63 | -3.48 | 14.76 |
| Year: 1989 | Fixed effect of year | -2.28 | 0.55 | -2.73 | 18.17 |
| Year: 1990 | Fixed effect of year | -2.30 | 0.59 | -2.54 | 9.72 |
| Year: 1991 | Fixed effect of year | -2.67 | 0.59 | -3.00 | 11.76 |
| Year: 1992 | Fixed effect of year | -2.49 | 0.66 | -2.84 | 13.03 |
| Year: 1993 | Fixed effect of year | -2.56 | 0.71 | -2.95 | 13.81 |
| Year: 1994 | Fixed effect of year | -3.21 | 0.82 | -3.69 | 13.92 |
| Year: 1995 | Fixed effect of year | -3.43 | 0.73 | -3.91 | 13.13 |
| Year: 1996 | Fixed effect of year | -2.45 | 0.81 | -2.95 | 18.56 |
| Year: 1997 | Fixed effect of year | -2.61 | 0.91 | -3.02 | 14.64 |
| Year: 1998 | Fixed effect of year | -4.03 | 0.91 | -4.45 | 9.96 |
| Year: 1999 | Fixed effect of year | -2.68 | 0.79 | -3.15 | 16.20 |
| Year: 2000 | Fixed effect of year | -1.97 | 0.74 | -2.52 | 24.25 |
| Year: 2001 | Fixed effect of year | -0.73 | 0.63 | -1.22 | 50.38 |
| Year: 2002 | Fixed effect of year | -0.75 | 0.54 | -1.24 | 49.26 |
| Year: 2003 | Fixed effect of year | 0.71 | 0.52 | 0.42 | 49.84 |
| Year: 2004 | Fixed effect of year | -0.79 | 0.56 | -0.85 | 7.99 |
| Year: 2005 | Fixed effect of year | -0.31 | 0.45 | -0.78 | 87.40 |
| Year: 2006 | Fixed effect of year | -0.76 | 0.53 | -1.10 | 35.84 |
| Year: 2007 | Fixed effect of year | -1.10 | 0.51 | -1.63 | 39.07 |
| Year: 2008 | Fixed effect of year | -0.49 | 0.53 | -1.07 | 73.67 |
| Year: 2009 | Fixed effect of year | -0.28 | 0.49 | -0.90 | 106.00 |
| Year: 2010 | Fixed effect of year | -0.44 | 0.46 | -0.76 | 53.94 |
| Year: 2011 | Fixed effect of year | 0.23 | 0.45 | -0.20 | 3531.90 |
| Year: 2012 | Fixed effect of year | -0.33 | 0.44 | -0.66 | 65.47 |
| Year: 2013 | Fixed effect of year | 0.46 | 0.46 | 0.07 | 148.09 |
| Year: 2014 | Fixed effect of year | 0.40 | 0.44 | 0.16 | 87.42 |
| Year: 2015 | Fixed effect of year | 0.11 | 0.41 | -0.09 | 1635.48 |
| Year: 2016 | Fixed effect of year | 0.48 | 0.41 | 0.30 | 46.73 |
| Year: 2017 | Fixed effect of year | 0.58 | 0.41 | 0.43 | 29.57 |
| Year: 2018 | Fixed effect of year | 0.66 | 0.39 | 0.28 | 80.07 |
| Year: 2019 | Fixed effect of year | -0.30 | 0.40 | -0.71 | 82.40 |
| Year: 2020 | Fixed effect of year | 0.42 | 0.42 | 0.05 | 154.87 |
| Year: 2021 | Fixed effect of year | 1.56 | 0.42 | 1.33 | 15.84 |
| Year: 2022 | Fixed effect of year | 0.88 | 0.41 | 0.52 | 52.22 |
| range | Matérn range | 79.55 | 5.37 | 79.86 | 0.39 |
| $\sigma_{\omega}$ | marginal standard deviation of the spatial field | 19.51 | 2.59 | 19.29 | 1.10 |
| $\sigma_{\varepsilon}$ | marginal standard deviation of spatiotemporal field | 10.57 | 0.88 | 10.64 | 0.66 |


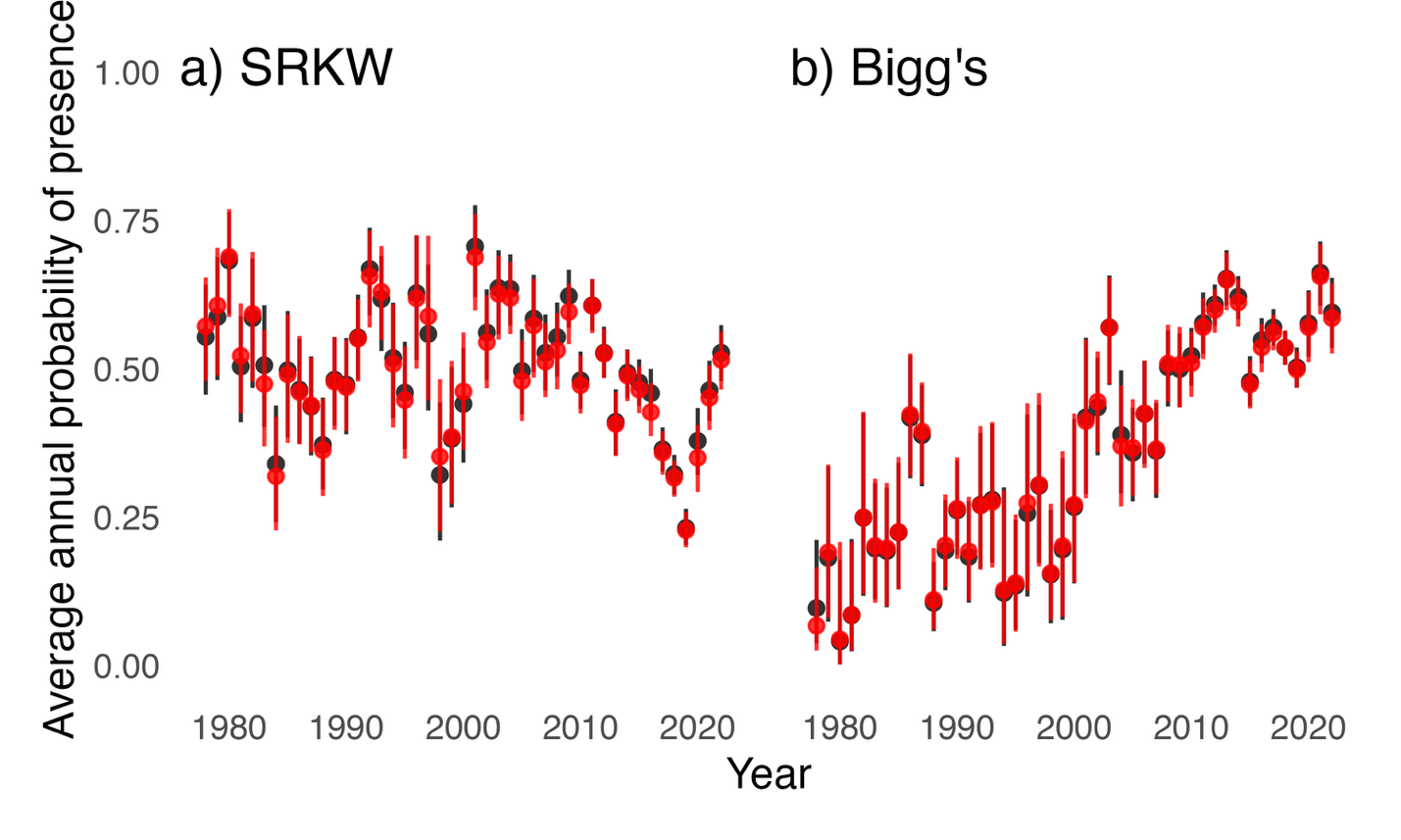


**Fig 2.**  **Average predicted probability of presence (points) and 95% prediction intervals (errorbars) across all quadrants in each year for SRKW (a) and Bigg’s (b) for models with all data sources included (black) and models without hydrophone data (red).**


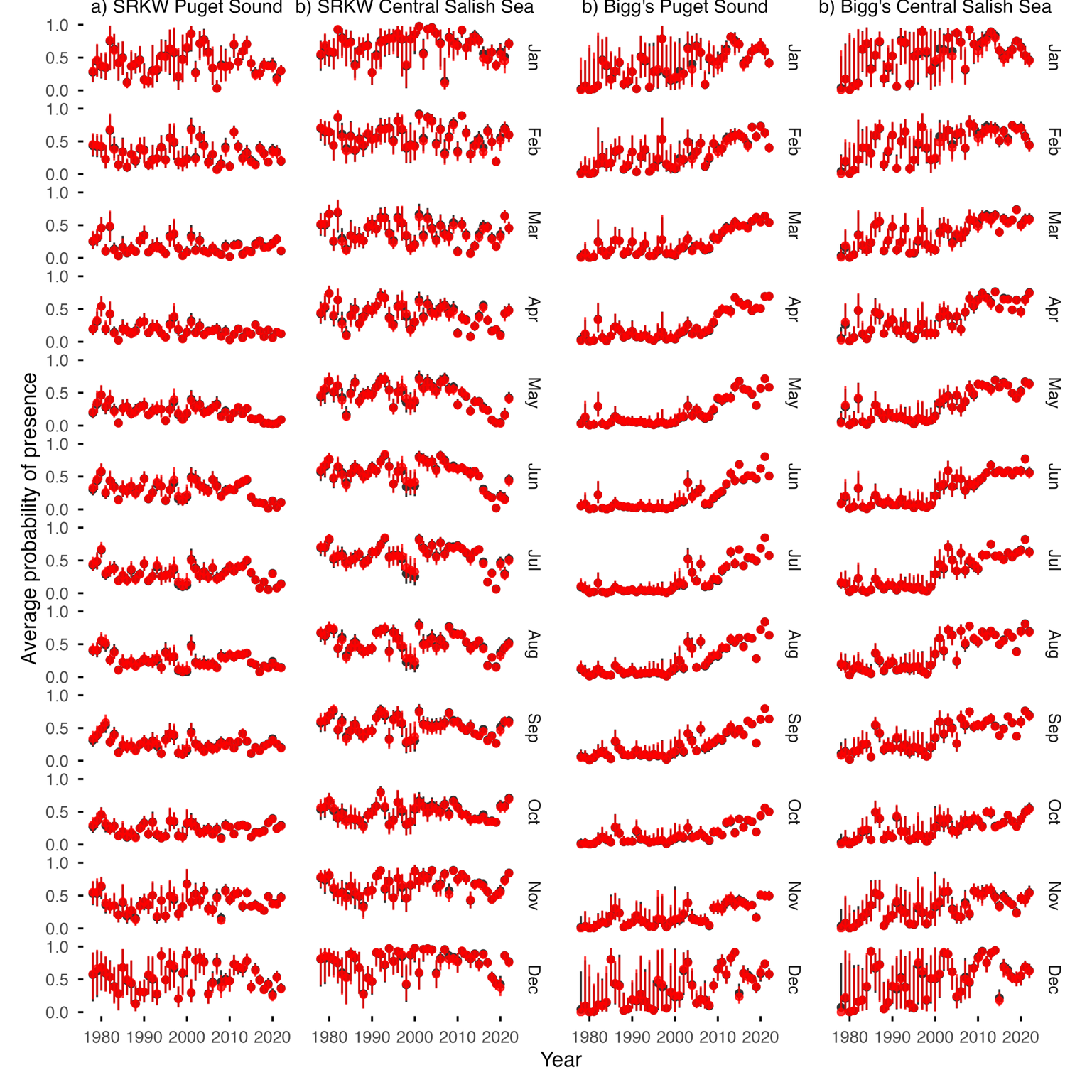


**Fig 3.**  **Average monthly predicted probability of presence (points) and 95% prediction intervals (errorbars) for SRKW (a-b) and Bigg’s (c-d) for models with all data sources included (black) and models without hydrophone data (red).**
