# Supplementary material for: Increasing presence of Bigg’s killer whales and changing seasonality of Southern Resident killer whales in Washington waters
Source: PLoS One. 2026 Jun 24;21(6):e0350181. doi: 10.1371/journal.pone.0350181 (PMC13293393; doi:10.1371/journal.pone.0350181)
Supplement: Attachment — Submitted filename: Response to reviewers.docx [file pone.0350181.s003.docx]

**Response to reviewers on PONE-D-26-01639: "Increasing presence of Bigg's killer whales and changing seasonality of Southern Resident killer whales in Washington waters”**

**Academic Editor**

Thank you for submitting your manuscript to PLOS ONE. After careful consideration, we feel that it has merit but does not fully meet PLOS ONE’s publication criteria as it currently stands. Therefore, we invite you to submit a revised version of the manuscript that addresses the points raised during the review process.

Thank you for taking the time to review our manuscript. We present here a revised version of the manuscript as well as a point-by-point response to all comments.

We received two sets of reviewer comments. Both reviewers find the manuscript valuable and methodologically sound. The primary revisions requested involve expanding the ecological and management interpretation of the results, particularly with respect to environmental change, prey dynamics, and inter-ecotype interactions. Additional clarifications and minor edits have also been suggested. No major methodological revisions appear to be required at this stage.

We have made all requested reviewer changes as indicated below. Line numbers refer to the tracked changes version of the text.

However, I believe the manuscript would benefit from clearer reporting of the sensitivity of the results to data types (acoustic versus visual detections). The supplementary material seems to suggest the method was sensitive to the data types. I suggest moving the sensitivity analysis to the main text and discussing it. If the conclusions do differ "meaningfully" between data types, the manuscript may warrant additional review by a methodological expert.

Thank you for taking the time to review the sensitivity tests. The results presented in the supplementary material may have been confusing. While the individual parameter estimates did differ between models, the resulting distributions over space and time did not differ very much. We have changed the style of figure 2 and added an additional figure and text to the supplement (File S1) to demonstrate this as well as added the following text to the main manuscript: “However, since results did not greatly differ from those of the base model, results from this sensitivity test are presented in the appendix (S1 File).” (ln 179-181).

File S2: Figure 2:

**Fig 2.**  Average predicted probability of presence (points) and 95% prediction intervals (errorbars) across all quadrants in each year for SRKW (a) and Bigg’s (b) for models with all data sources included (black) and models without hydrophone data (red).

File S1 Figure 3:

**Fig 3.** Average monthly predicted probability of presence (points) and 95% prediction intervals (errorbars) for SRKW (a-b) and Bigg’s (c-d) for models with all data sources included (black) and models without hydrophone data (red).

**Reviewer 1**

This paper created species distribution models for two ecotypes of killer whale, southern resident killer whales (SRKW) and Bigg’s killer whales using the Whale Museum’s Sighting Archive. These models showed different long term trends in either ecotype, with SRKW presence variable over time, but a recent decrease in presence during the summer, and Bigg’s presence generally increasing over time.

This paper is valuable for its use of a historical data set which was able to provide long term trends. The authors did a good job handling bias in sighting data in the way they developed the absence points for their models. Work of this type is valuable for creating both a knowledge base and tracking change for killer whale management.

Generally, this work is well done, and I don’t have many overarching comments or concerns. The abstract and introduction of the paper highlight the management applications but I think management applications could be further developed in the discussion. I would like to see the authors discuss more about environmental change; spatial models like this can lend themselves so well to robust environmental/habitat use conclusions. Have there been notable changes in the region over the past 4-5 decades? How could environmental changes have affected the presence of these killer whale ecotypes? What might this mean for the future? For the decline in SRKW?

Thank you for taking the time to review our manuscript and for your thoughtful comments. We have added more information to the discussion about environmental change as suggested. Please see our more detailed comments below.


Specific comments

Line 59: You allude to changes in the timing of salmon presence in this section. It would be interesting to bring this back in the discussion in a section that discusses environmental change. Environmental links to changes in salmon return? What does this mean for killer whale management? It would be good to round out the story.

Thank you for this feedback. We have added an additional paragraph to the discussion which addresses environmental changes and links to salmon returns (lines 435-449):

“In addition, environmental covariates could be added to the model as salmon demographics may be linked to changing climate. Quinn and Losee (54)found that Salish Sea residential salmonids have declined and are smaller than non-resident fish, and this could be tied to changes in environmental conditions. Southern Residents prefer larger salmonids[55] and therefore this may cause a decrease in their occurrence in winter months when larger, non-residential salmon have migrated outside of Salish Sea to coastal waters[54]. Other research has shown a decline in Chinook size and age across the U.S. West Coast [56], therefore, less biomass for Southern Residents to consume. Additionally, Puget Sound Chinook were found to have high exposure and sensitivity to changing environmental conditions compared to certain other salmonid populations [57], and temperature and salinity are increasing in Puget Sound while dissolved oxygen is declining[58]. As previously mentioned, Stewart et al. (13)found that shifts in Southern Resident timing in Salish sea may be related to Fraser Chinook abundance, and these fish are vulnerable to environmental conditions[59]. Fraser Chinook have also experienced a decline in size at age that may be linked to environmental variables [60] and more lipid-rich Fraser runs have declined [61], which the authors note could be tied to ocean conditions.”

Line 64: Is there information available on overall population trends for Biggs killer whales? Could that be related to an increase near Washington? Lay these things out for readers that are not familiar.

We have added a sentence to the introduction which discusses possible increase in population size in the region (lines 68-71). It reads:

“Though photo-identification effort for Bigg’s killer whales has been ongoing since 1974, abundance estimates are rare. However, photo-identification studies suggest that populations have been increasing by 3-4% per year since 2010[16,17].”

Line 67: Citation for increase in pinnipeds in the region?
Great catch! We have added a citation to this line (line 73).

Line 70: Add an approximation of the length of the time series here, it will be good for comparison to your long term data.

We have added the length of the time series in previous studies as suggested (line 74).

Line 267: How many SRKW observations had pod designations? How many observations of each pod?

We have added this information as suggested in line 287-288.

Line 308: Capitalize Inlet and Basin

We have made this change as suggested throughout the text.

Line 373: Could you delve a little bit more here into some of the literature on interactions between killer whale populations/ecotypes? A sentence or two on what has been observed elsewhere and what increased interaction could mean for these two groups?

We have added a few sentences to the discussion about this as suggested (lines 420-424):

“This may positively impact SRKW, by releasing more salmonid prey for SRKW consumption. However, it may also displace SRKW from their primary habitat, leading to interaction with other fish-eating killer whales (such as the Northern resident killer whales) [51].”

Line 380: I like the mention of future models including prey and covariates. I would love a bit of discussion on a potential trophic cascade model, incorporating the effects of Biggs predation on the competition between pinnipeds and SRKW.

This is a great idea for a future model. We have added the following sentence to the discussion to address this (lines 432-434):

“A future joint model of both SRKW and Bigg’s, incorporating prey-specific covariates, could allow for a deeper understanding of interactions between these two ecotypes, the effects of Bigg’s predation on pinnipeds, and resulting pinniped consumption of salmon.”

**Reviewer 2**

This manuscript presents a thorough and well-executed analysis of long-term changes in the occurrence of Southern Resident and Bigg’s killer whales in Washington waters. The study leverages an impressive multi-decadal dataset from The Whale Museum’s sightings archive. The authors apply a species distribution modeling framework to both ecotypes, allowing for direct comparison of trends and overlap. The results are clearly presented. The documented increase in Bigg’s killer whale presence, combined with pod-specific declines in Southern Residents’ occurrence, provides important ecological and management insights. Overall, the manuscript is well written, logically structured, and makes a meaningful contribution to understanding changing predator dynamics in the area.

Thank you for taking the time to review our manuscript. We appreciate your thoughtful suggestions and have responded to your comments in more detail below.

I have only minor comments aimed at improving clarity:

L. 44. “which are a fish-eating population of 73 individuals in 2024” – maybe “were” rather than “are”? 2024 was two years ago, and there were actually 75 two months ago.

We have made this change as suggested.

L. 90-99. This section would benefit from a clearer formulation of the study’s objective. Rather than only describing what was done, it should more explicitly explain why the work was undertaken and what overarching purpose or research question the study aims to address.

We have rearranged and re-worked this paragraph so our questions are more clear. It now begins (lines 99-102):

“In this study, we investigate how SRKW and Bigg’s presence has changed in Washington waters over time (1978-2022). We also compare how SRKW and Bigg’s habitat use has changed relative to each other, which may have implications for how these ecotypes interact with each other.”

L. 164-167. In fact, detection distance depends not only on the frequency and source level of the vocalization, but also on several additional factors, such as the depth of the source and the hydrophone, the bathymetry and bottom substrate between them, water temperature and salinity structure, background noise levels, and other environmental conditions.

We have added these specifics to the text (ln 183-184).

L. 193. Is “location” the same thing as a “quadrant” here?

It does. Good catch! We have changed this to refer to quadrant instead of location (ln 212).

L. 234-238. On which scale it was done – in quadrants or larger?

We specified further down in the text that this was calculated for each year, month, and quadrant but we agree that it makes sense to specify earlier on so we’ve added that to line 254.

Finally, I find the Discussion somewhat underdeveloped. As a reader, I would appreciate a deeper exploration of the potential drivers behind the observed patterns, particularly the pod-specific changes in occurrence. Expanding on possible ecological, behavioral, or management-related explanations would make the discussion more engaging and insightful.

We have added an additional paragraph to the discussion to add more detail to possible differences between pods that could lead to the patterns we see in our study (lines 374-382):

“This continued overall presence of SRKW is largely driven by J-pod, since K- and L-pod presence has declined since 2016 (Fig. 5). There is some evidence that specific Chinook stocks are of variable importance to the different pods [12] and diet varies across pods [46] which could help to explain variation in presence between the pods. Social and cultural learning likely also play an important role in killer whale habitat use and foraging behavior. The strength of social cohesion between members of each pod may be influenced by environmental factors, such as prey availability, as well as population size [47]. Furthermore, individuals and particular matrilines may also play an important role in social cohesion [48]. Differences in the social cohesion among pods may contribute to the variation in presence seen in our study.”
