# Supplementary material for: Increasing presence of Bigg’s killer whales and changing seasonality of Southern Resident killer whales in Washington waters
Source: PLoS One. 2026 Jun 24;21(6):e0350181. doi: 10.1371/journal.pone.0350181 (PMC13293393; doi:10.1371/journal.pone.0350181)
Supplement: Attachment — Submitted filename: Response to reviewers rev2.docx [file pone.0350181.s004.docx]

**Response to reviewers on PONE-D-26-01639: "Increasing presence of Bigg's killer whales and changing seasonality of Southern Resident killer whales in Washington waters”**

**Academic Editor**

While the manuscript has been substantially improved and the revisions are generally responsive to the concerns raised by the reviewers and me, a few minor items must be addressed before I can make a final decision.

Thank you for taking the additional time to review our manuscript. We have addressed all your comments below. The line numbers refer to the tracked changes version of the manuscript.

First, the sensitivity analysis comparing models with and without hydrophone data remains insufficiently described in the main text. Although the authors state that results did not differ greatly between models, I had requested a more substantive treatment of this analysis. Please include one or two sentences in the main text providing specific numerical comparisons between the full and no-hydrophone models rather than relying solely on a qualitative description (“did not greatly differ” sounds very subjective).

We have changed the text in the methods so it now reads (lines 177-178): “As a sensitivity, additional models were run without the acoustic data and more details about this sensitivity test can be found in the appendix (S1 File).” In addition, we have added the following specifics to the results section:

Line 260-262: “As a sensitivity, models were also run excluding acoustic detections. This resulted in 127 fewer days of SRKW presence (2.0 % of days with SRKW presence) and 68 fewer days of Bigg’s presence (1.6 % of days with Bigg’s presence; S1 File).”

Line 275-279: “The models without acoustic data resulted in a < 3 % difference in almost all average predicted probabilities of presence across years and ecotypes. Additionally, the confidence intervals of these predictions from both models overlapped, suggesting there was no difference in the predicted annual pattern when this data was included or excluded (S1 File).”

Line 311-314: “The models without acoustic data resulted in a < 5 % difference in almost all average predicted probabilities of presence across months, years, regions, and ecotypes. Additionally, the confidence intervals of the predictions from both models overlapped, suggesting there was no difference in predicted monthly patterns when this data was included or excluded (File S1).”

Second, two references cited in the new environmental change paragraph (Mascarenas et al. and Peacock et al.) are listed as an in-review manuscript and a preprint, respectively. Please confirm their current publication status and, if they remain unpublished, explicitly note their preliminary nature in the text so readers can appropriately evaluate the strength of evidence.

Peacock et al. (ref 59) and Mascarenas et al (ref 58) are still in pre-print, so we have added the following qualifications to the discussion:

Lines 424-429: “Additionally, Puget Sound Chinook were found to have high exposure and sensitivity to changing environmental conditions compared to certain other salmonid populations [57], and preliminary results suggest temperature and salinity are increasing in Puget Sound while dissolved oxygen is declining [58]. As previously mentioned, Stewart et al. (13) found that shifts in Southern Resident timing in Salish sea may be related to Fraser Chinook abundance, and preliminary analyses suggest these fish are vulnerable to environmental conditions [59].”

Third, please correct the minor typographical error “killingof” in the Discussion before resubmission.

We have made this change as suggested.
