# Supplementary material for: Increasing presence of Bigg’s killer whales and changing seasonality of Southern Resident killer whales in Washington waters
Source: PLoS One. 2026 Jun 24;21(6):e0350181. doi: 10.1371/journal.pone.0350181 (PMC13293393; doi:10.1371/journal.pone.0350181)
Supplement: Peer review history

# Peer review: Decision Letter 0

**DOI:** 10.1371/journal.pone.0350181.r001

**Authors:** Masami Fujiwara

22 Feb 2026

PONE-D-26-01639

Increasing presence of Bigg's killer whales and changing seasonality of Southern Resident killer whales in Washington waters

PLOS One

Dear Dr. Rand,

Thank you for submitting your manuscript to PLOS ONE. After careful consideration, we feel that it has merit but does not fully meet PLOS ONE’s publication criteria as it currently stands. Therefore, we invite you to submit a revised version of the manuscript that addresses the points raised during the review process.

We received two sets of reviewer comments. Both reviewers find the manuscript valuable and methodologically sound. The primary revisions requested involve expanding the ecological and management interpretation of the results, particularly with respect to environmental change, prey dynamics, and inter-ecotype interactions. Additional clarifications and minor edits have also been suggested. No major methodological revisions appear to be required at this stage.

However, I believe the manuscript would benefit from clearer reporting of the sensitivity of the results to data types (acoustic versus visual detections). The supplementary material seems to suggest the method was sensitive to the data types. I suggest moving the sensitivity analysis to the main text and discussing it. If the conclusions do differ "meaningfully" between data types, the manuscript may warrant additional review by a methodological expert.

Please submit your revised manuscript by Apr 08 2026 11:59PM. If you will need more time than this to complete your revisions, please reply to this message or contact the journal office at plosone@plos.org. When you're ready to submit your revision, log on to https://www.editorialmanager.com/pone/ and select the 'Submissions Needing Revision' folder to locate your manuscript file.

Please include the following items when submitting your revised manuscript:

- A letter that responds to each point raised by the academic editor and reviewer(s). You should upload this letter as a separate file labeled 'Response to Reviewers'.
- A marked-up copy of your manuscript that highlights changes made to the original version. You should upload this as a separate file labeled 'Revised Manuscript with Track Changes'.
- An unmarked version of your revised paper without tracked changes. You should upload this as a separate file labeled 'Manuscript'

-->If you would like to make changes to your financial disclosure, please include your updated statement in your cover letter. Guidelines for resubmitting your figure files are available below the reviewer comments at the end of this letter.

If applicable, we recommend that you deposit your laboratory protocols in protocols.io to enhance the reproducibility of your results. Protocols.io assigns your protocol its own identifier (DOI) so that it can be cited independently in the future. For instructions see: https://journals.plos.org/plosone/s/submission-guidelines#loc-laboratory-protocols. Additionally, PLOS ONE offers an option for publishing peer-reviewed Lab Protocol articles, which describe protocols hosted on protocols.io. Read more information on sharing protocols at https://plos.org/protocols?utm_medium=editorial-email&utm_source=authorletters&utm_campaign=protocols.

We look forward to receiving your revised manuscript.

Kind regards,

Masami Fujiwara, PhD

Academic Editor

PLOS One

Journal Requirements:

When submitting your revision, we need you to address these additional requirements.

1. Please ensure that your manuscript meets PLOS ONE's style requirements, including those for file naming. The PLOS ONE style templates can be found at

https://journals.plos.org/plosone/s/file?id=wjVg/PLOSOne_formatting_sample_main_body.pdf and

https://journals.plos.org/plosone/s/file?id=ba62/PLOSOne_formatting_sample_title_authors_affiliations.pdf

2. Please note that PLOS One has specific guidelines on code sharing for submissions in which author-generated code underpins the findings in the manuscript. In these cases, we expect all author-generated code to be made available without restrictions upon publication of the work. Please review our guidelines at https://journals.plos.org/plosone/s/materials-and-software-sharing#loc-sharing-code and ensure that your code is shared in a way that follows best practice and facilitates reproducibility and reuse.

3. Thank you for stating the following financial disclosure:

“This project was possible through the funding of the Puget Sound Partnership, with the support of the Puget Sound Partnership.

Z.R.R. was funded in part by a grant from Washington Sea Grant, University of Washington, pursuant to National Oceanic and Atmospheric Administration (Grant No. NA23OAR4170532).”

Please state what role the funders took in the study.  If the funders had no role, please state: "The funders had no role in study design, data collection and analysis, decision to publish, or preparation of the manuscript."

If this statement is not correct you must amend it as needed.

Please include this amended Role of Funder statement in your cover letter; we will change the online submission form on your behalf.

4. If the reviewer comments include a recommendation to cite specific previously published works, please review and evaluate these publications to determine whether they are relevant and should be cited. There is no requirement to cite these works unless the editor has indicated otherwise.

5. Please review your reference list to ensure that it is complete and correct. If you have cited papers that have been retracted, please include the rationale for doing so in the manuscript text, or remove these references and replace them with relevant current references. Any changes to the reference list should be mentioned in the rebuttal letter that accompanies your revised manuscript. If you need to cite a retracted article, indicate the article’s retracted status in the References list and also include a citation and full reference for the retraction notice.

[Note: HTML markup is below. Please do not edit.]

Reviewers' comments:

Reviewer's Responses to Questions

**Comments to the Author**

1. Is the manuscript technically sound, and do the data support the conclusions?

The manuscript must describe a technically sound piece of scientific research with data that supports the conclusions. Experiments must have been conducted rigorously, with appropriate controls, replication, and sample sizes. The conclusions must be drawn appropriately based on the data presented.

Reviewer #1: Yes

Reviewer #2: Yes

**********

2. Has the statistical analysis been performed appropriately and rigorously?

Reviewer #1: Yes

Reviewer #2: Yes

**********

3. Have the authors made all data underlying the findings in their manuscript fully available?

The PLOS Data policy requires authors to make all data underlying the findings described in their manuscript fully available without restriction, with rare exception (please refer to the Data Availability Statement in the manuscript PDF file). The data should be provided as part of the manuscript or its supporting information, or deposited to a public repository. For example, in addition to summary statistics, the data points behind means, medians and variance measures should be available. If there are restrictions on publicly sharing data—e.g. participant privacy or use of data from a third party—those must be specified.

Reviewer #1: No

Reviewer #2: No

**********

4. Is the manuscript presented in an intelligible fashion and written in standard English?

PLOS ONE does not copyedit accepted manuscripts, so the language in submitted articles must be clear, correct, and unambiguous. Any typographical or grammatical errors should be corrected at revision, so please note any specific errors here.

Reviewer #1: Yes

Reviewer #2: Yes

**********

5. Review Comments to the Author

Please use the space provided to explain your answers to the questions above. You may also include additional comments for the author, including concerns about dual publication, research ethics, or publication ethics. (Please upload your review as an attachment if it exceeds 20,000 characters)

Reviewer #1: Review for PONE-D-26-01639 “Increasing presence of Bigg's killer whales and changing seasonality of Southern Resident killer whales in Washington waters”

This paper created species distribution models for two ecotypes of killer whale, southern resident killer whales (SRKW) and Bigg’s killer whales using the Whale Museum’s Sighting Archive. These models showed different long term trends in either ecotype, with SRKW presence variable over time, but a recent decrease in presence during the summer, and Bigg’s presence generally increasing over time.

This paper is valuable for its use of a historical data set which was able to provide long term trends. The authors did a good job handling bias in sighting data in the way they developed the absence points for their models. Work of this type is valuable for creating both a knowledge base and tracking change for killer whale management.

Generally, this work is well done, and I don’t have many overarching comments or concerns. The abstract and introduction of the paper highlight the management applications but I think management applications could be further developed in the discussion. I would like to see the authors discuss more about environmental change; spatial models like this can lend themselves so well to robust environmental/habitat use conclusions. Have there been notable changes in the region over the past 4-5 decades? How could environmental changes have affected the presence of these killer whale ecotypes? What might this mean for the future? For the decline in SRKW?

Specific comments

Line 59: You allude to changes in the timing of salmon presence in this section. It would be interesting to bring this back in the discussion in a section that discusses environmental change. Environmental links to changes in salmon return? What does this mean for killer whale management? It would be good to round out the story.

Line 64: Is there information available on overall population trends for Biggs killer whales? Could that be related to an increase near Washington? Lay these things out for readers that are not familiar.

Line 67: Citation for increase in pinnipeds in the region?

Line 70: Add an approximation of the length of the time series here, it will be good for comparison to your long term data.

Line 267: How many SRKW observations had pod designations? How many observations of each pod?

Line 308: Capitalize Inlet and Basin

Line 373: Could you delve a little bit more here into some of the literature on interactions between killer whale populations/ecotypes? A sentence or two on what has been observed elsewhere and what increased interaction could mean for these two groups?

Line 380: I like the mention of future models including prey and covariates. I would love a bit of discussion on a potential trophic cascade model, incorporating the effects of Biggs predation on the competition between pinnipeds and SRKW.

Reviewer #2: This manuscript presents a thorough and well-executed analysis of long-term changes in the occurrence of Southern Resident and Bigg’s killer whales in Washington waters. The study leverages an impressive multi-decadal dataset from The Whale Museum’s sightings archive. The authors apply a species distribution modeling framework to both ecotypes, allowing for direct comparison of trends and overlap. The results are clearly presented. The documented increase in Bigg’s killer whale presence, combined with pod-specific declines in Southern Residents’ occurrence, provides important ecological and management insights. Overall, the manuscript is well written, logically structured, and makes a meaningful contribution to understanding changing predator dynamics in the area.

I have only minor comments aimed at improving clarity:

L. 44. “which are a fish-eating population of 73 individuals in 2024” – maybe “were” rather than “are”? 2024 was two years ago, and there were actually 75 two months ago.

L. 90-99. This section would benefit from a clearer formulation of the study’s objective. Rather than only describing what was done, it should more explicitly explain why the work was undertaken and what overarching purpose or research question the study aims to address.

L. 164-167. In fact, detection distance depends not only on the frequency and source level of the vocalization, but also on several additional factors, such as the depth of the source and the hydrophone, the bathymetry and bottom substrate between them, water temperature and salinity structure, background noise levels, and other environmental conditions.

L. 193. Is “location” the same thing as a “quadrant” here?

L. 234-238. On which scale it was done – in quadrants or larger?

Finally, I find the Discussion somewhat underdeveloped. As a reader, I would appreciate a deeper exploration of the potential drivers behind the observed patterns, particularly the pod-specific changes in occurrence. Expanding on possible ecological, behavioral, or management-related explanations would make the discussion more engaging and insightful.

**********

6. PLOS authors have the option to publish the peer review history of their article (what does this mean?). If published, this will include your full peer review and any attached files.

If you choose “no”, your identity will remain anonymous but your review may still be made public.

**Do you want your identity to be public for this peer review?** For information about this choice, including consent withdrawal, please see our Privacy Policy.

Reviewer #1: No

Reviewer #2: No

**********

[NOTE: If reviewer comments were submitted as an attachment file, they will be attached to this email and accessible via the submission site. Please log into your account, locate the manuscript record, and check for the action link "View Attachments". If this link does not appear, there are no attachment files.]

To ensure your figures meet our technical requirements, please review our figure guidelines: https://journals.plos.org/plosone/s/figures

You may also use PLOS’s free figure tool, NAAS, to help you prepare publication quality figures: https://journals.plos.org/plosone/s/figures#loc-tools-for-figure-preparation.

NAAS will assess whether your figures meet our technical requirements by comparing each figure against our figure specifications.

# Author comment: Author response to Decision Letter 1

**DOI:** 10.1371/journal.pone.0350181.r002

30 Apr 2026

Thank you for taking the time to review our manuscript. We have attached a full response letter that includes point by point responses to your comments as a file with this submission.

Attachment Submitted filename: Response to reviewers.docx

# Editor report: Decision Letter 1

**DOI:** 10.1371/journal.pone.0350181.r003

**Authors:** Masami Fujiwara

8 May 2026

PONE-D-26-01639R1

Increasing presence of Bigg's killer whales and changing seasonality of Southern Resident killer whales in Washington waters

PLOS One

Dear Dr. Rand,

Thank you for submitting your manuscript to PLOS ONE. After careful consideration, we feel that it has merit but does not fully meet PLOS ONE’s publication criteria as it currently stands. Therefore, we invite you to submit a revised version of the manuscript that addresses the points raised during the review process.

While the manuscript has been substantially improved and the revisions are generally responsive to the concerns raised by the reviewers and me, a few minor items must be addressed before I can make a final decision. First, the sensitivity analysis comparing models with and without hydrophone data remains insufficiently described in the main text. Although the authors state that results did not differ greatly between models, I had requested a more substantive treatment of this analysis. Please include one or two sentences in the main text providing specific numerical comparisons between the full and no-hydrophone models rather than relying solely on a qualitative description (“did not greatly differ” sounds very subjective). Second, two references cited in the new environmental change paragraph (Mascarenas et al. and Peacock et al.) are listed as an in-review manuscript and a preprint, respectively. Please confirm their current publication status and, if they remain unpublished, explicitly note their preliminary nature in the text so readers can appropriately evaluate the strength of evidence. Third, please correct the minor typographical error “killingof” in the Discussion before resubmission.

Please submit your revised manuscript by Jun 22 2026 11:59PM. If you will need more time than this to complete your revisions, please reply to this message or contact the journal office at plosone@plos.org. When you're ready to submit your revision, log on to https://www.editorialmanager.com/pone/ and select the 'Submissions Needing Revision' folder to locate your manuscript file.

Please include the following items when submitting your revised manuscript:

- A letter that responds to each point raised by the academic editor and reviewer(s). You should upload this letter as a separate file labeled 'Response to Reviewers'.
- A marked-up copy of your manuscript that highlights changes made to the original version. You should upload this as a separate file labeled 'Revised Manuscript with Track Changes'.
- An unmarked version of your revised paper without tracked changes. You should upload this as a separate file labeled 'Manuscript'.

If you would like to make changes to your financial disclosure, please include your updated statement in your cover letter. Guidelines for resubmitting your figure files are available below the reviewer comments at the end of this letter.

If applicable, we recommend that you deposit your laboratory protocols in protocols.io to enhance the reproducibility of your results. Protocols.io assigns your protocol its own identifier (DOI) so that it can be cited independently in the future. For instructions see: https://journals.plos.org/plosone/s/submission-guidelines#loc-laboratory-protocols. Additionally, PLOS ONE offers an option for publishing peer-reviewed Lab Protocol articles, which describe protocols hosted on protocols.io. Read more information on sharing protocols at https://plos.org/protocols?utm_medium=editorial-email&utm_source=authorletters&utm_campaign=protocols.

As the corresponding author, your ORCID iD is verified in the submission system and will appear in the published article. PLOS supports the use of ORCID, and we encourage all coauthors to register for an ORCID iD and use it as well. Please encourage your coauthors to verify their ORCID iD within the submission system before final acceptance, as unverified ORCID iDs will not appear in the published article. *Only* the individual author can complete the verification step; PLOS staff *cannot* verify ORCID iDs on behalf of authors.

We look forward to receiving your revised manuscript.

Kind regards,

Masami Fujiwara, PhD

Academic Editor

PLOS One

Journal Requirements:

1. If the reviewer comments include a recommendation to cite specific previously published works, please review and evaluate these publications to determine whether they are relevant and should be cited. There is no requirement to cite these works unless the editor has indicated otherwise.

2. Please review your reference list to ensure that it is complete and correct. If you have cited papers that have been retracted, please include the rationale for doing so in the manuscript text, or remove these references and replace them with relevant current references. Any changes to the reference list should be mentioned in the rebuttal letter that accompanies your revised manuscript. If you need to cite a retracted article, indicate the article’s retracted status in the References list and also include a citation and full reference for the retraction notice.

[Note: HTML markup is below. Please do not edit.]

[NOTE: If reviewer comments were submitted as an attachment file, they will be attached to this email and accessible via the submission site. Please log into your account, locate the manuscript record, and check for the action link "View Attachments". If this link does not appear, there are no attachment files.]

To ensure your figures meet our technical requirements, please review our figure guidelines: https://journals.plos.org/plosone/s/figures

You may also use PLOS’s free figure tool, NAAS, to help you prepare publication quality figures: https://journals.plos.org/plosone/s/figures#loc-tools-for-figure-preparation.

NAAS will assess whether your figures meet our technical requirements by comparing each figure against our figure specifications.

# Author comment: Author response to Decision Letter 2

**DOI:** 10.1371/journal.pone.0350181.r004

8 May 2026

A letter addressing all comments from the academic editor has been attached.

Attachment Submitted filename: Response to reviewers rev2.docx

# Editor report: Decision Letter 2

**DOI:** 10.1371/journal.pone.0350181.r005

**Authors:** Masami Fujiwara

12 May 2026

Increasing presence of Bigg's killer whales and changing seasonality of Southern Resident killer whales in Washington waters

PONE-D-26-01639R2

Dear Dr. Rand,

We’re pleased to inform you that your manuscript has been judged scientifically suitable for publication and will be formally accepted for publication once it meets all outstanding technical requirements.

Within one week, you’ll receive an e-mail detailing the required amendments. When these have been addressed, you’ll receive a formal acceptance letter and your manuscript will be scheduled for publication.

An invoice will be generated when your article is formally accepted. Please note, if your institution has a publishing partnership with PLOS and your article meets the relevant criteria, all or part of your publication costs will be covered. Please make sure your user information is up-to-date by logging into Editorial Manager at Editorial Manager® and clicking the ‘Update My Information' link at the top of the page. For questions related to billing, please contact billing support.

If your institution or institutions have a press office, please notify them about your upcoming paper to help maximize its impact. If they’ll be preparing press materials, please inform our press team as soon as possible -- no later than 48 hours after receiving the formal acceptance. Your manuscript will remain under strict press embargo until 2 pm Eastern Time on the date of publication. For more information, please contact onepress@plos.org.

Kind regards,

Masami Fujiwara, PhD

Academic Editor

PLOS One

Additional Editor Comments (optional):

Reviewers' comments:

# Editor report: Acceptance letter

**DOI:** 10.1371/journal.pone.0350181.r006

**Authors:** Masami Fujiwara

PONE-D-26-01639R2

PLOS One

Dear Dr. Rand,

I'm pleased to inform you that your manuscript has been deemed suitable for publication in PLOS One. Congratulations! Your manuscript is now being handed over to our production team.

At this stage, our production department will prepare your paper for publication. This includes ensuring the following:

* All references, tables, and figures are properly cited

* All relevant supporting information is included in the manuscript submission,

* There are no issues that prevent the paper from being properly typeset

You will receive further instructions from the production team, including instructions on how to review your proof when it is ready. Please keep in mind that we are working through a large volume of accepted articles, so please give us a few days to review your paper and let you know the next and final steps.

Lastly, if your institution or institutions have a press office, please let them know about your upcoming paper now to help maximize its impact. If they'll be preparing press materials, please inform our press team within the next 48 hours. Your manuscript will remain under strict press embargo until 2 pm Eastern Time on the date of publication. For more information, please contact onepress@plos.org.

You will receive an invoice from PLOS for your publication fee after your manuscript has reached the completed accept phase. If you receive an email requesting payment before acceptance or for any other service, this may be a phishing scheme. Learn how to identify phishing emails and protect your accounts at https://explore.plos.org/phishing.

If we can help with anything else, please email us at customercare@plos.org.

Thank you for submitting your work to PLOS ONE and supporting open access.

Kind regards,

PLOS ONE Editorial Office Staff

on behalf of

Dr. Masami Fujiwara

Academic Editor

PLOS One
